# Nuclear mechanobiology rules immune cells’ functions: from differentiation to cell trafficking and pathogen killing

**DOI:** 10.1080/19491034.2025.2590843

**Published:** 2026-01-06

**Authors:** Jörg Renkawitz, Allen Yesin, Janina Kroll, Aidan T. Cabral, Sarah D’Annunzio, Hawa Racine Thiam

**Affiliations:** a Biomedical Center, Walter Brendel Center of Experimental Medicine, Institute of Cardiovascular Physiology and Pathophysiology, Klinikum der Universität, Ludwig Maximilians Universität München, Munich, Germany; b Department of Bioengineering, Stanford University, Stanford, CA, USA; c Department of Microbiology and Immunology; d Sarafan ChEM-H Institute, Stanford University, Stanford, CA, USA; e Biohub, San Francisco, CA, USA

**Keywords:** Cell migration, chromatin, cytoskeleton-to-nucleus coupling, immune cells, lamins, NETosis, nuclear condensates, nuclear deformation and shape

## Abstract

The immune system functions within tissue microenvironments of mechanical and geometrical constraints. Within these constraints, immune cells must rapidly move and execute effector functions to regulate innate and adaptive immunity. Here, we review the impact of nuclear mechanobiology on immune cell functionality. We define how non-genetic physical properties of the nucleus such as shape, stiffness and deformability are regulated and directly impact immune cell functions ranging from trafficking routes to pathogen killing. We highlight that studying immune cells allowed breakthroughs in understanding how the nucleus acts as a sensor for spatial constraints, as a break or enabler for cell migration, and as an extracellular trap to kill pathogens. Further, we discuss the unknowns of nuclear mechanobiology and consider the impact of chromatin, condensates, and nuclear membrane components. Together, this review provides an overarching framework of the cellular, physical, and immunological principles of nuclear mechanobiology in immune cells.

## GRAPHICAL ABSTRACT

**Figure:**
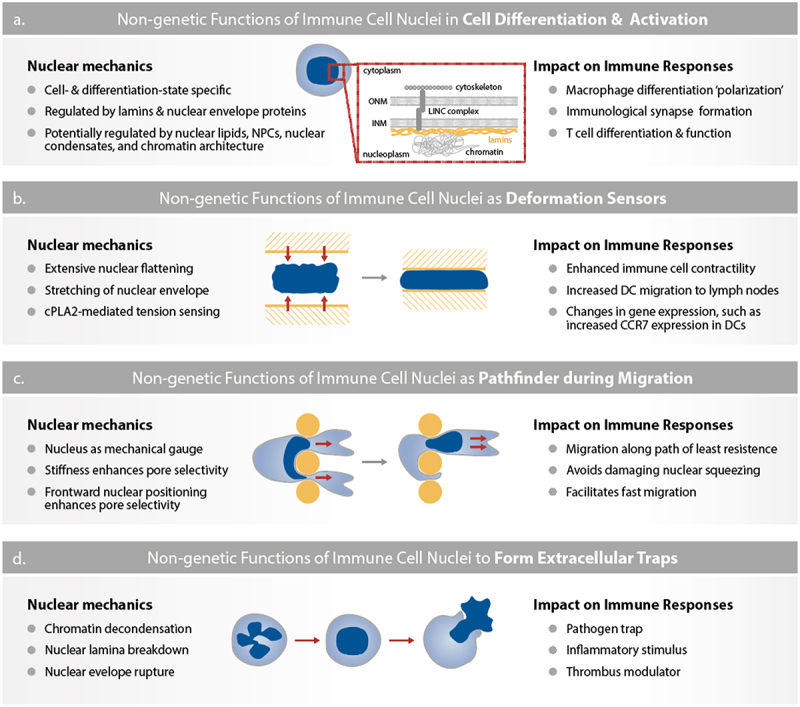

## Introduction

The nucleus is a defining feature of eukaryotes, where it stores the genome and regulates gene expression. However, the nucleus is also a large and physically complex organelle. In mammalian somatic cells, nuclear diameter ranges between 5 and 20 µm [1] and the nucleus can occupy up to 80% of the cell volume [2]. Furthermore, distinct nuclear structures such as chromatin, nuclear lamina, and the nuclear membrane [3] provide the nucleus with physical and mechanical properties, such as shape, size, stiffness, viscoelasticity, deformability, and integrity [4]. These physical properties define the nucleus’s ability to sense, withstand and respond to mechanical forces [5]. Moreover, cells can actively alter nuclear composition, thereby changing the mechanical properties of the nucleus, making it an active material that can adapt over time to the physicochemical properties of the intra- and extracellular space [4]. While many foundational principles of nuclear mechanobiology have been identified in cell culture models and nonimmune cell types [4], studies in highly dynamic immune cells have revealed novel insights and concepts, such as the role of the nucleus as a cellular deformation sensor [6,7], pathfinder [8], regulator of cell migration [9], and generator of extracellular traps (ETs) [10]. During these processes, the nucleus and its content play a critical non-genetic role. Given the key discoveries made in recent years, we propose that recognizing the critical role of nuclear mechanics in immune cell response is one of the breakthroughs in immunology over the past 20 years.

The goal of an immune response is to maintain homeostasis by protecting the host from pathogens and other damaging agents. This task requires immune responses to be deployed rapidly at minute time scales. Such rapid responses are achieved through cooperation of the immune system’s cellular and humoral branches, which allows the rapid detection and elimination of pathogens and injured cells and the restoration of homeostasis [11,12]. In multicellular systems, immune cells that constitute the cellular branch of the immune system [12] must solve a physical problem: how to rapidly reach and achieve their functions in tissues > 1000s of cell lengths distant from their initial location? In adult vertebrates, most immune cells are produced in the bone marrow but must function in any tissue [13,14]. Reaching these distant tissues requires immune cells to squeeze in, travel through, and squeeze out of the blood and lymph systems [15–17]. Once in tissues, immune cells must navigate and find their targets in tissue environments with various packing densities, stiffness and viscoelasticity [18], before deploying a specific response to eliminate the target and/or restore homeostasis [14]. Additionally, in lymphatic organs such as the cell-dense lymph nodes, antigen-presenting cells like dendritic cells and adaptive immune cells like T cells constantly move to achieve proximity between cells carrying appropriate antigen/antigen-receptor pairs [19–21]. In summary, many immune cells are constantly moving through diverse and complex tissue environments to mount effective immune responses. Considering the fast time scales of these responses and the physical challenges immune cells encounter in tissues, it is not surprising that the mechanical properties of immune cells or their intracellular contents, like the nucleus, play a critical role in immune responses.

While many underlying aspects of the mechanobiology of immune responses remain to be discovered, key principles have been identified. For instance, several reviews discussed how the physical properties of tissues, such as stiffness, topography, and ECM architecture, regulate immune cell behavior [22–24]; how mechanical forces regulate immune cell interactions such as during the formation of immunological synapses [25] or target cell killing by cytotoxic lymphocytes [26]; and how immune cells sense and generate mechanical forces and how these forces regulate immune cell activation, intracellular signaling and migration [27]. Immune cells use mechanisms similar to nonimmune cells to sense and respond to extracellular mechanical stimuli. For instance, they sense external mechanical forces through activation of mechanosensitive surface proteins such as ion channels (piezo 1), or membrane receptors (integrin or immune cell-specific mechanosensitive T and B cell receptors) [27]. Activation of these surface receptors initiates intracellular signaling cascades and secondary messengers such as calcium, which alter the organization and dynamics of the cytoskeleton. The cytoskeleton itself and its regulators, like the actin nucleator Arp2/3, are mechanosensitive and can directly transduce mechanical forces [28]. At the level of the nucleus, extracellular mechanical forces are often transmitted through the ‘Linker of Nucleoskeleton and Cytoskeleton’ (LINC) complex, a network of proteins that physically connects the nucleus to the cytoskeleton [29–31]. However, the nucleus can also directly sense mechanical forces independently of the LINC complex, for instance, during cell migration under confinement [9,16]. While the role of surface proteins and the cytoskeleton in immune cell mechanosensitivity has been extensively reviewed [22–24,32], we currently lack a comprehensive review on how nuclear mechanics regulate immune function. This review aims to close this gap by discussing our current understanding of how nuclear mechanics regulates immune cell behavior and how immune cells, in turn, actively modify the mechanical properties of the nucleus to achieve their roles in host defense. On the functional level, we will discuss how nuclear mechanics actively regulates migration in confining environments, pathfinding, immune cell activation, differentiation, and pathogen killing. Together, this knowledge positions nuclear mechanobiology (the mechanical properties and functions of the nucleus) as an active regulator of immune responses.

## Nuclear shapes and mechanics regulate immune cell trafficking

Unlike other cell types that primarily degrade the extracellular matrix to facilitate movement in dense matrices, many immune cells rely on ameboid migration [33,34]. This migration strategy allows them to traverse confined spaces and move through micron-sized pores as small as 1–2 µm within tissues, across endothelial barriers, and through dense tumor microenvironments without proteolysis [16,35]. Given the long distances these cells travel, and the large number of immune cells present in organisms like humans, this non-proteolytic migration mode is crucial for immune surveillance, pathogen clearance, and tissue repair, all while keeping tissue microenvironments intact. However, each immune cell contains a nucleus that can be up to 10 times stiffer than the rest of the cell [36]. In B cells, the nucleus can take various shapes and occupy up to 80% of the cell volume [2]. As a result, the size and deformability – the ability to change shape while maintaining mechanical integrity – of the nucleus dictates a cell’s ability to pass through narrow pores smaller than the diameter of the nucleus [9,37,38]. If the nucleus cannot deform sufficiently, the cell must either (i) find an alternative route, (ii) risk damaging its nucleus, or (iii) risk becoming immobilized. In the following subsections, we will describe how nuclear shapes and other mechanical properties of the nucleus, along with mechanical forces generated by immune cells, help immune cells traverse confined spaces while preserving the overall integrity of the nucleus.

### Nuclear shapes and their role in immune cell trafficking

**Figure 1.**
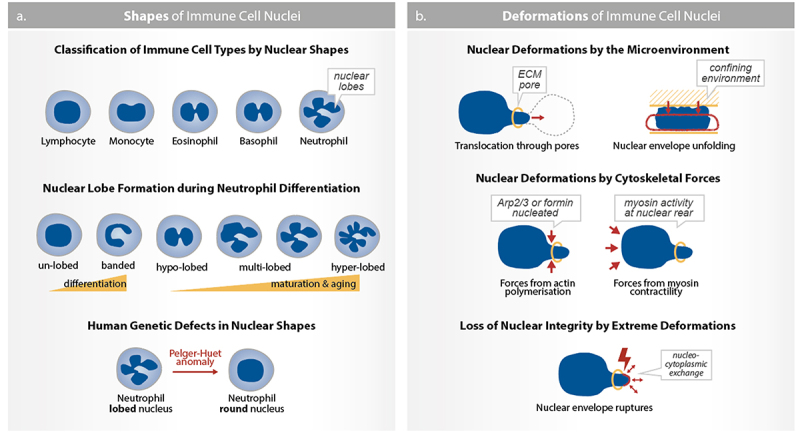
Shapes and deformations of immune cell nuclei. (a) Immune cell nuclei can be classified by shape, ranging from round shapes in lymphocytes, over dented shapes in monocytes, to segmented shapes in eosinophils, basophils, and neutrophils. In neutrophils, the nuclear shape develops during differentiation (from un-lobed to banded) and maturation (from hypo-lobed to multi-lobed) which occur in the bone marrow. Mature neutrophils with multilobed nuclei are released into the blood circulation where they increase their nuclear lobes over time leading to hyper-lobed neutrophils. Notably, patients with Pelger-Huët anomaly carrying mutations in the lamin B receptor (LBR) have neutrophils with non-segmented round nuclei. (b) In addition to their given shape, immune cell nuclei undergo substantial deformations during their movement in confining environments, such as through narrow ECM pores in the microenvironment, driven by forces from actin polymerisation (Arp2/3 or formin) at the cell front and myosin contractility at the cell rear. Most extremely, these deformations can cause the rupture of the nuclear envelope leading to nucleo-cytoplasmic exchange.

A defining characteristic of immune cells is the distinct morphology of their nuclei, which can take an array of shapes, ranging from oval or invaginated in T cells to multilobulated in neutrophils (Figure 1(a)). For decades, this morphology of the nucleus has been a key parameter for differentiating between types of immune cells and nonimmune cells in blood and tissue samples such as in histopathological sections [39,40]. Importantly, nuclear morphologies in immune cells are not static but dynamically change over different timescales. For example, the nucleus transitions from a bilobulated to a round shape during monocyte differentiation to macrophages over the course of days [41]. During dendritic cells and neutrophils migration through a narrow pore, the nucleus transitions from a round or multilobulated to a dumbbell shape on a timescale of minutes [8,9,37]. Further, alterations in the nuclear morphology of immune cells are associated with diseases. For example, neutrophils in patients with Pelger-Huët anomaly exhibit fewer nuclear lobes (hyposegmentation), whereas deficiencies in vitamin B12, folic acid or lipid metabolic enzyme, as well as infection with Helicobacter pylori, lead to an increased number of nuclear lobes (hypersegmentation) in neutrophils [42,43].

While the characteristics of these morphological features are well established, the molecular basis and functional roles of different nuclear shapes in immune responses are just at the beginning of being explored [44]. The lamin B receptor (LBR) is critical for forming the segmented nuclear morphology of neutrophils and is mutated in patients with Pelger-Huët anomaly (see also Lipids in nuclear mechanics.). Further, microtubules and intermediate filaments are involved in shaping the neutrophil’s nucleus. In HL60-derived neutrophils and primary human neutrophils, the microtubule-organizing center (MTOC) is located close to nuclear lobes, often nestled between them and adjacent to nuclear indentations which are sensitive to microtubule depolymerization [8,45,46]. Similarly, the intermediate filament vimentin is enriched at concave regions of the nucleus in mouse neutrophils, and vimentin knockout cells have fewer nuclear lobes [47]. In immune cells with non-segmented nuclei, the proximity of the MTOC to the nucleus also correlates with formation of a nuclear indentation, such as in B cells forming an immunological synapse [48] and in dendritic cells migrating through complex environments [8,49]. Together, this data suggests that microtubules and intermediate filaments exert forces onto the nucleus, likely through the LINC complex (see section “The nuclear envelope as a protective elastic shell”), potentially leading to deformations of immune cell nuclei.

**Figure 2.**
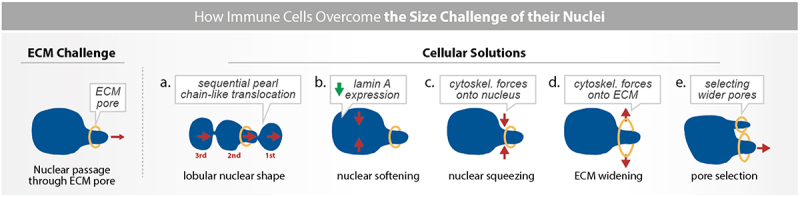
Nuclear mechanics as a bottleneck for immune cell trafficking. During their lifetime, immune cells frequently migrate. This includes their trafficking (i) into tissues and lymphoid organs upon their initial differentiation in the bone marrow, (ii) through tissues during their explorative patrolling to detect pathogens and other deviations from homeostasis, (iii) and into infection and inflammation sites, (iv) as well as through lymphoid organs during the execution of innate and adaptive immune responses. On these routes, immune cells cross crowded and confining microenvironments, such as narrow pores in the extracellular environment (ECM; orange). Moving the nucleus through these confining environments represents a physical challenge for cells, given the size and stiffness of the nucleus. Immune cells evolved at least 5 principles to solve this challenge, which are not mutually exclusive but can complement each other: (a) subdividing the nuclear size into smaller packages by the segmentation into nuclear lobes, leading to sequential pearl chain-like translocation through the ECM pore, (b) softening the nucleus by downregulating the expression of lamin A, (c) squeezing the nucleus by forces from the actin and myosin cytoskeletons, (d) widening the environmental pore by drilling the nucleus into the pore and by the cytoskeleton pushing against the ECM obstacle perpendicularly to the migration direction, and (e) by mechanical gauging for the size of close-by pores by using the nucleus as a mechanical probe, followed by the translocation of the nucleus and the entire cell body through the wider pore.

One intriguing question is why most immune cells have non-ovoid nuclear shapes. Studies from neutrophils and T cells suggest that nuclear shape is important for immune cell deformability and development (see section “Nuclear shapes and mechanics regulate immune cell development and activation”). In neutrophils, nuclear multilobulation is proposed to increase nuclear deformability, potentially by subdividing the nucleus into multiple smaller lobes (Figure 1(b) and Figure 2(a)). Indeed, neutrophils with multilobed nuclei can deform to pass through much smaller pores than other immune cells (down to 1 µm in diameter [9,50]). Primary human neutrophils that have more nuclear lobes ( > 3 lobes), along with high CD16 (FcRIII) and CD62L (L-Selectin) expression, migrate faster through confined microchannels compared to neutrophils with fewer (1–2) nuclear lobes [50]. However, neutrophils increase the number of nuclear lobes as they differentiate (Figure 1(a)), making it challenging to distinguish whether differences in migration behavior arise from nuclear shape or from other biochemical differences between progenitors and differentiated neutrophils. Thus, it remains unclear whether the lobular shape itself or the differentiation state facilitates neutrophil passage through narrow pores. In a study using HL60-derived neutrophils, knocking down LBR resulted in a round nucleus, but did not impact the cell’s ability to deform through 3–5 µm pores [51]. However, when the authors overexpressed lamin A/C, the nucleus became round, and cells slowed their passage through 3–5 µm pores [51]. This suggests that the stiffness of the nucleus (proportional to the amount of lamin A/C [9] rather than its shape may be the limiting factor in the migration speed through narrow pores. Nonetheless, it remains unresolved if nuclear multilobulation provides a migratory advantage to neutrophils and other granulocytes that must frequently traverse dense tissues to accomplish their host defense functions [16,52].

The shape of immune cell nuclei is not only a defining feature that distinguishes different immune cell types but can also be actively modified by forces from the surrounding microenvironment (Figure 1(b)). Large and rapid nuclear deformation down to 3 µm leads to unfolding of the nuclear envelope, which triggers a mechanosensing response in immature dendritic cells as well as in cancer and Zebrafish progenitor cells [6,53]. This nuclear compression activates cytosolic phospholipase 2 (cPLA2; lipolytic enzyme that catalyze the production of arachidonic acid; recently reviewed in [54]), increases the overall cell contractility, and is linked to the initiation of a fast and persistent amoeboid-like migration in a variety of cells, including epithelial, mesenchymal, cancer, and immune cells [55,56]. In vivo, this shape-sensing mechanism induces the expression of the chemokine receptor CCR7 in DCs, facilitating the migration of skin DCs to lymph nodes during steady state, without prior pathogen encounter [7]. These studies suggest that nuclear deformations caused by a confining microenvironment lead to changes in cytoskeletal dynamics and gene expression, which in turn impact the migration strategy adopted by immune cells. From a mechanical perspective, it will be fascinating to understand how the differing mechanical properties and shapes of immune cell nuclei influence the extent of these cytoskeletal and transcriptional responses caused by nuclear deformation. Further, it will be valuable to investigate these phenomena in diverse physiological and pathophysiological tissues with varying mechanical properties, such as stiffness and geometry.

### Nuclear deformability regulates immune cell migration under confinement

For immune cells to migrate *in vivo*, they must deform their 5–20 µm diameter and 1–5 kPa stiff nucleus [57] through the tight pores of the basement membranes or the 1–20 µm pores of interstitial tissues [37] (Figure 1(b)). For instance, live imaging in *Drosophila* or *Medeka* fish demonstrated that leukocyte nuclei undergo frequent and reversible cycles of deformation in vivo [9,58]. But how do immune cells overcome the challenge of nuclear deformation during confined migration?

The first strategy immune cells employ to overcome the limitation of nuclear deformability – independent of nuclear morphology as discussed in the previous section – is having a softer, more deformable nucleus [59]. Softer nuclei have been linked to faster cell migration in nonimmune cells [37,38], a more rapid immune cells transit through non-degradable 3D collagen matrices [37], and quicker passage through micron-scale pores [51] (Figure 2(b)). Indeed, nuclear stiffness determines the speed and probability of immune cells passing through pores smaller than 50% of the nuclear diameter at minute timescales [9,37,51]. In *Drosophila*, immune cells experience drastic nuclear deformation when they transition from the less-constrained environment of the wing to the more-constrained environment of the wing ‘vessel’ [58]. These immune cells adapt to the increasing constrain of the in vivo environment by increasing nuclear deformability through overexpression of more elastic B-type lamins (see section “The nuclear envelope as a protective elastic shell”), facilitating efficient navigation through narrow tissue architecture [58]. These studies show that nuclear stiffness directly regulates nuclear deformability and imposes a physical limitation to immune cell migration [16,60,61]. In later sections, we will discuss more extensively how immune cells regulate nuclear stiffness and overall deformability.

When the intrinsic deformability of the nucleus is too low, immune cells generate intracellular forces from the cytoskeleton to actively deform their nuclei (Figure 2(c)). For instance, immature dendritic cells squeeze their nuclei through micron-sized pores by assembling an Arp2/3 nucleated perinuclear actin network, which compresses the nucleus [9,62]. T cells employ actin polymerization by Formin-like 1 (FMNL1) to squeeze their nuclei through confining environments [63]. Beyond forces onto the nucleus by the polymerization of actin, mature dendritic cells generate a myosin II-based contractility machinery to selectively squeeze their nuclei through narrow pores [64,65]. Similarly, T cells rely on myosin contractility to squeeze their nucleus through the endothelial barrier during trans-endothelial migration (TEM) [66,67], and neutrophils rely on the activity of the unconventional class I myosin 1f to squeeze their nucleus through physical barriers [68]. Although most studies investigated individual cytoskeleton force generators in individual cell types and different physical environments, a general picture emerges: actin polymerization perpendicular to the direction of cell migration squeezes the nucleus while rearward myosin contractility pushes the nucleus through the constricting pore. Importantly, the cytoskeleton can act not only as a deformer of the nucleus but also as a ‘protector.’ For instance, DOCK8, a protein whose mutation causes immunodeficiencies [69], regulates a mechanosensitive actin pool in dendritic cells and T cells that shields the nucleus and facilitates migration under confinement [70,71].

In certain extreme cases where immune cells cannot deform their nucleus to migrate under confinement, they can use their nucleus (in addition to the cytoskeleton) [72,73] to deform the extracellular environment (Figure 2(d)). T cells and neutrophils insert nuclear lobes into the endothelium to displace the dense cytoskeletal network of endothelial cells and transmigrate through the endothelium [74]. Furthermore, in dense environments, where even the largest pores impair free passage, leukocytes switch polarity by positioning their nucleus behind the centrosome and other organelles [71]. This mesenchymal configuration enables the assembly of a central F-actin pool between the cell front and nucleus, which pushes outward to transiently dilate a path for organelles and the nucleus [71].

Computational models have provided further insights into how immune cells overcome the challenge of nuclear deformation during confined migration. Multi-compartment computational models that allow independent control of cell and nuclear deformability suggest both must be optimized for effective cell migration through pores smaller than the nuclear diameter. Both too-soft and too-stiff cells impair migration through narrow pores, with too-soft cells being defective at directional migration, while too-stiff cells failing at deforming through pores [75]. Consistent with published experimental data, stiff nuclei impair the cell’s ability to enter and rapidly transit through small pores. Although this computational study focused on nonimmune cells, expanding it to immune cells suggests that nuclear stiffness is optimized for migration through confined spaces [75]. Continuum elastic models have been used to calculate the force field required to deform the nucleus during immune cell migration under confinement [62]. These models predicted the experimentally observed actin polymerization around the nucleus of dendritic cells migrating through narrow pores [9], which exerts lateral forces that squeeze the sides of the nucleus as it enters constrictions. While these models provide valuable predictions, their main limitation lies in capturing the dynamic nature of nuclear deformation processes that occur on rapid timescales during confined migration. Furthermore, these models lack comparative experimental data of the mechanical properties of immune cell nuclei since many mechanical measurements of the nucleus are either done in nonimmune cells, in isolated nuclei or through the plasma membrane and the underlying actin cortex (see Table 1). This presents a challenge in fully understanding the real-time interplay between nuclear mechanics and immune cell dynamics.

**Table 1.** Summary of quantitative tools used to assess the mechanical properties and functions of the nucleus in cells.

Tool	Application	Limitation
Micropipette aspiration; reviewed in [235,236]	Records cell deformation upon pressure suction to measure time-dependent creep behavior, tension, Young’s modulus/stiffness, and viscoelasticity of the nucleus. It can be used to apply stress on the nucleus and measure the nuclear response to understand which components of the nucleus (lamins, membrane proteins, chromatin) contribute to its mechanical properties and how nuclear stretching impacts chromatin organization [141,237–240].	Low throughput (~10 min for 1 measurement), size and geometry of the mouth of glass capillary micropipette are critical for accuracy and sensitivity. Must consider the contribution of the cytoskeleton requiring removal of the cytoskeleton or isolation of the nucleus if cytoskeleton is coupled. Micropipette aspiration of nuclei cannot be interpreted using conventional methods and requires a model for nuclear stability in pipette.
Atomic Force Microscopy (AFM); reviewed in [241]	Measures cantilever deflection during indentation of the whole cell or isolated nucleus. Local mechanics can be probed with a sharp tip, while global properties can be assessed with a tipless cantilever [241,242]. From cantilever deformation, mechanical properties such as Young’s modulus (stiffness) and the viscous modulus (time-dependent behavior) can be quantified [198,237,241]. In isolated nuclei, AFM can capture power-law rheology and creep behavior, revealing stiff responses at short timescales and softening over longer timescales [237]. Enables nanomechanical and force mapping to generate spatially resolved elasticity/viscoelasticity maps, which can be correlated with structural information to visualize nuclear topography and mechanical heterogeneity [243,244]. Can also be used to apply well-defined compressive forces on the nucleus and assess the impact of these forces on cell state and function [6].	In intact cells, the cytoskeleton masks the nuclear response, so measurements require cytoskeleton removal, which perturbs the system. Similarly, isolated nuclei may show different mechanical properties than nuclei inside intact cells. Measurements of nuclei within intact cells require adhesive cells. However, multiple immune cells like mature dendritic cells are only loosely adhesive, requiring “glueing” the cells to the surface (e.g., via poly-L-lysine), which may impact on the cellular behaviors. Measurements are low throughput, with a single probe measuring one cell at a time.
Optical/magnetic tweezers; reviewed in [245]	Optical tweezers can be used to indent the nuclear periphery and measure the corresponding response of the nucleus and cell [246]. Membrane stretching experiments (trap opposite sides of nucleus) to measure its elastic and viscoelastic properties. Optical tweezers allow to perform microrheology by oscillating trapped beads to obtain frequency-dependent storage/loss modulus [245]. Magnetic tweezers allow the assessment of nuclear stiffness and how the nuclear and subnuclear compartment such as the lamina network respond to mechanical forces [147].	Low throughput and must be performed on isolated nuclei unless an object with high refractive index (e.g., beads) is introduced in cells or intracellular vesicles can be trapped and used. Must consider photothermal and phototoxicity effects. Optical tweezers are highly sensitive and are thus best for small force ranges such as 1-100pN. Most tweezers require calibration which can be long and challenging.
Microfluidic devices: microchannels; cell confiner, microposts/pillars; reviewed in [247–249]	Assessment of nuclear deformability in live cells by measuring transit times and rates through narrow channels or microposts; nuclear deformability governs how quickly a nucleus squeezes through constrictions [9,250,251]. Can also be applied to determine how nuclear mechanics impacts pathfinding during cell migration [49,9]. High-throughput microfluidic micropipette aspiration can quantify nuclear elastic and viscoelastic properties like Young’s modulus and viscous modulus of intact cells using the Jeffreys rheological model [252]. Confined migration through microchannels induces quantifiable changes in nuclear shape; the dynamics of nuclear envelope proteins and nuclear envelope rupture; allowing correlation between nuclear architecture, integrity, and mechanical behavior under confinement [9,76,77,253]. Contactless microfluidic systems such as the cell confiner [254,255] allow to study the impact of well-controlled unidirectional confinement on nuclear mechanics and cell functions such as migration and proliferation [55,256,257]; confined cells can be collected for further biochemical and omics studies [7,254]. Newer contactless cell confiner can apply a (10–10^3^ µN) range of compressive forces to suspended cells and monitor nuclear structural responses, like changes in Lamin A/C or chromatin organization, showing how the nucleus adapts to load over time [258]. Microfluidic methods are high throughput, allowing assessment of 100s of cells at the same time, such as during the migration through hundreds of parallel microchannels [9,254,255,259].	Measurements are mostly qualitative; nuclear mechanics are inferred from transit times or deformations rather than directly quantified. Constrictions and forces applied may not perfectly mimic physiological conditions. For instance, PDMS-based microchannels provide a non-deformable cellular microenvironment. And compressive forces from the cell confiner are much higher than what cells experience in vivo
3D hydrogels (collagen matrices) and transwells; reviewed in [52,249]	Allow to assess nuclear deformability by measuring the efficiency of cell migration through transwell pores or dense collagen networks [37,38]. Collagen matrices can mimic the stiffness and geometrical complexity of the in vivo environment [37,52]. Transwell assays allow to assess the impact of nuclear deformation on genome integrity and gene expression; cells can be collected for further biochemical and omics-based assays [38,143,260].	Transwell assays are not compatible with live microscopy making it difficult to measure the dynamics of nuclear deformation. Collagen matrices are heterogeneous with a range of pore sizes and fiber thickness making it difficult to model and predict the physical challenge imposed on embedded cells. The complexity of collagen matrices makes it difficult to distinguish nuclear from cell entanglement.
Biosensors/reporters	**Genetically encoded** Lamin A/C tension sensor: FRET-based construct that reports tension across lamin filaments in live cells [261]. cPLA2: relocalizes to stretched nuclear membranes, used as a readout of nuclear membrane tension [165]. ALPIN (Amphipathic Lipid Packing sensor domain Inside the Nucleus): biosensor designed to monitor the lipid packing and tension of the nuclear membrane [133].	Restricted to genetically tractable cells for FRET-based sensors; many primary immune cells are difficult/impossible to transfect. Dye-based probes are indirect, reporting on lipid packing or order rather than whole-nucleus mechanics. Lower temporal resolution compared to mechanical assays. Assumes a single tagged protein or lipid property reflects the full nuclear mechanical state.
	**Dye-based** Flipper-TR, a fluorescent probe [262] that inserts into membranes and reports lipid order/tension through changes in fluorescence lifetime, was used to measure nuclear membrane tension [198].	
Brillouin microscopy; reviewed in [263].	A non-contact method that relies on Brillouin light scattering that can characterize local micromechanical properties including viscoelastic properties and longitudinal moduli of the nucleus [264,265].	Brillouin microscopy is highly directional and does not measure Young’s Modulus. Must know local refractive index to process data. Low sensitivity to soft materials which may apply to specific immune cells (i.e., neutrophil). Low temporal resolution due to long acquisition times may contribute to phototoxicity of sensitive immune cells.
Particle tracking micro rheology; reviewed in [266].	Track thermal or force-induced motion of fluorescent beads or genetically encoded multimeric (GEM) nanoparticles to calculate viscoelastic properties (storage and loss modulus) of nucleoplasm with high spatiotemporal resolution. This gives insights into the physical properties of the milieu but also its crowding [267–269].	Beads must be microinjected into the nucleus since the membrane coating of beads delivered by endocytosis might alter measurements. These beads might also not reach the nucleus. GEMs with nuclear localization signals are genetically encoded which makes their delivery and use challenging for short-lived immune cells such as neutrophils. Must be able to differentiate active vs passive motion of particles. Requires reasonably small particles (<1µm).

In summary, immune cells have evolved at least three mechanisms to overcome the challenge of nuclear deformation during confined migration: they (i) harbor more deformable nuclei, (ii) actively deform the nucleus by generating physical forces mostly via the cytoskeleton or (iii) use the nucleus and the cytoskeleton to deform the surrounding tissue environment and facilitate efficient migration through dense environments (Figure 2).

Immune cells use various mechanisms to highly deform their nuclei and migrate through narrow pores, but how do they survive such extensive nuclear deformation? Dendritic cells and monocytes migrating in narrow pores (below 2 µm in pore size) can rupture their nuclear envelope [76,77] (Figure 1(b)). Interestingly, computational modeling indicates that transient nuclear ruptures, by softening the nucleus, facilitate cell passage through narrow pores [75]. In dendritic cells, such ruptures trigger ESCRT-mediated nuclear membrane repair [76]. Immune cells with impaired nuclear repair mechanisms undergo rapid cell death after migration through narrow pores [76]. Thus, immune cells’ ability to rapidly and profoundly deform their nuclei while surviving and continuing to perform their functions provides a unique opportunity to study the design principles of a deformable nucleus. Additionally, recent findings showed that the non-membrane surrounded centrosome is vulnerable to mechanical deformation and breakage during the motility of immune cells [78]. Therefore, studying immune cells as a model with high spatiotemporal dynamics can help to uncover fundamental principles in the mechanobiology of organelles beyond just the nucleus.

### Nuclear mechanics regulate pathfinding by immune cells

Leukocytes need to navigate complex microenvironments composed of diverse guidance cues, including pores of various sizes and different chemokine gradients [16,79]. Many aspects of how leukocytes coordinate path decisions in these complex environments using receptor inputs and cytoskeletal remodeling have been identified [49,80–85], including how leukocytes sense guidance cues by probing their surrounding with multiple parallel protrusions [83,86]. Additionally, leukocytes employ their nucleus as a mechanical gauge to survey their local surroundings. To efficiently migrate through narrow gaps, leukocytes, such as dendritic cells, T cells, and neutrophils, use their nucleus to sample pore sizes and identify the path of least resistance [8] (Figure 2(e)). Enucleated dendritic cells are incapable of pore size selection [8]. In contrast, stiffening the nucleus by overexpressing a mutated form of lamin A enhances pore selectivity [8]. Pore size selection is ensured by positioning the nucleus at the front, allowing the cell to insert multiple nuclear protrusions into different pores. In the case of multilobulated nuclei of neutrophils, multiple lobes were observed probing pores in parallel [8]. Frontward positioning of the nucleus relies on actomyosin contractility but not the microtubule cytoskeleton, enabling dendritic cells and T cells to adapt their paths to competing guidance cues during navigation through complex environments [49]. However, how actomyosin generated forces are transmitted to the nucleus, and whether a physical connection to the cytoskeleton through the LINC complex is involved remains unclear (see extended discussion in reference [87]). Future work should address how nuclear mechanics, acting as a pathfinder, integrate with other signals (chemotactic receptor, positioning of the MTOC, and force generation through actin polymerization) to enable rapid and efficient decision making during immune cell migration.

## Nuclear shapes and mechanics regulate immune cell development, activation, and function

An effective immune response involves not only migration but also the ability of immune cells to detect danger signals (pathogenic or from inflammation), change their activation state and deploy an effector function such as proliferation, differentiation or killing. The decision-making process from signal detection to effector function requires biochemical modifications through gene expression, protein synthesis or post-translational modifications (PTMs). These biochemical modifications occur at a minute-to-hour time scale and are actively regulated by nuclear mechanobiology. Here, we will review the still limited yet exciting literature on the impact of nuclear mechanobiology in immune cell differentiation, activation, and pathogen killing.

### Nuclear shapes and mechanics regulate immune cell development and activation

The shape and composition of the nucleus, hence, its geometrical and mechanical properties, can actively control immune cell development, proliferation, and activation, suggesting that geometrical and mechanical remodelings of the nucleus are key catalysts and regulators of immune cell fate.

The importance of nuclear shape in immune cell development is supported by a recent study showing that the shape of naïve CD8^+^ T cell nucleus (spherical versus invaginated/banded) correlates with T cell proliferation and effector response upon activation [88]. Hale et al, performed high-throughput analysis of peripheral blood CD8^+^ T cells to discover a new subpopulation of naïve CD8^+^ T cells with nuclear envelope invaginations (i.e., non-spherical/banded nuclei) and perinuclear CD3 staining that constitutes 50% of the T cell pool in peripheral blood. Interestingly, this subpopulation of naïve CD8^+^ T cells with banded nuclei is reduced when T cells are cultured in vitro for 24 hours, suggesting their sensitivity to the physicochemical properties of the in vivo environment. Analysis of T cell development in mice revealed that most naïve CD8^+^ T cells display a banded nucleus architecture, and that these cells express higher levels of CD5, CXCR3 and NUR77, suggesting that naïve CD8^+^ T cells with banded nuclei have undergone stronger TCR signaling and selection during development, potentially explaining their enhanced survival during T cell maturation in the thymus. These CD8^+^ T cells with banded nuclei are the primary responders to antigen-loaded dendritic cells, further confirming their stronger TCR signaling compared to other subpopulations of peripheral blood CD8^+^ T cells. Upon TCR activation following antigen exposure, CD8^+^ T cells with banded nuclei display a stronger and longer increase in intracellular calcium, heightened proliferation and decreased expression of TCF1, suggesting that they preferentially differentiate into effector T cells [88]. Together, these studies show that the architecture of the nucleus is a critical driver of T cell development, proliferation and effector functions. Future mechanistic studies will be important to understand how nuclear shape impact T cell fate and functions. Along with nuclear shape, the composition of the nucleus regulates immune cell fate. For instance, lamin A/C, a nuclear envelope protein that modulates nuclear stiffness [89] (see section “The nuclear envelope as a protective elastic shell”), is critical for proper immune cell development and activation. Analysis of the lymphoid organs of Lmna^−/−^ mice demonstrated the important role of lamin A/C in the development of T and B cells. Lmna^−/−^ mice have reduced thymus and spleen size [90,91] and an age-dependent decrease in the number of cells constituting these lymphoid organs. Further, T cells’ positive selection in the thymus was disrupted, resulting in CD4 and CD8 T cells with reduced expression of TCRβ. Additionally, despite an increased number of CD4^+^ and CD8^+^ T cells in the spleen, the overall number of CD4^+^ and CD8^+^ T cells, as well as B cells, was severely decreased in Lmna^−/−^ mice [91]. Interestingly, the authors showed that placing Lmna^−/−^ T cells into Lmna^+/+^ host mice restored their development and functionality suggesting that the defects in T and B cell development in Lmna^−/−^ mice are immune cell-extrinsic effects potentially related to the rapidly degrading organs of Lmna^−/−^ mice [91].

From this study emerged the model that lamin A/C expression level does not impact T cell function [91]. However, recent studies have challenged this model, demonstrating that lamin A/C expression in CD4^+^ T cells regulates T cell activation and differentiation. In two seminal papers, Gonzalez-Granado et al. used quantitative cell and molecular biology approaches to show that lamin A/C is required for CD4^+^ T cell activation [92] and differentiation [93]. The authors showed that Lmna^−/−^ mice exhibit decreased T cell-mediated immune response in a model of CHS inflammatory response. Mechanistically, CD4^+^ T cells from Lmna^−/−^ mice have reduced expression of activation markers CD69 and CD25 following TCR stimulation. Upon TCR activation through antigen presentation, CD4^+^ T cells increase lamin A/C expression, which is required to efficiently form an immunological synapse between T cells and antigen-presenting cells, accelerate TCR clustering and enhance ERK1/2 signaling. Importantly, disrupting the LINC complex impairs the lamin A/C-mediated T cell activation, suggesting that a proper nuclear envelope architecture is required for T cell activation [92]. Using the same model of Lmna^−/−^ mice, the authors showed that lamin A/C is necessary for the differentiation of CD4^+^ T cells into T helper cells, which are critical for immunity against infection [94]. Indeed, naïve Lmna^−/−^ CD4^+^ T cells exhibit reduced IFNγ expression, which is a key driver of Th1 differentiation – compared to wild-type (WT) CD4^+^ T cells, suggesting a potential role of lamin A/C in Th1 differentiation. Consistently, Lmna^−/−^ CD4^+^ T cells cultured in vitro in Th1 or Th2 polarizing cytokines failed to differentiate into Th1 T cells without impacting Th2 differentiation. These findings suggest that lamin A/C, and potentially nuclear stiffness, selectively influences Th1 differentiation and activation [93].

In human macrophages, lamin A/C is phosphorylated, fragmented, and degraded early in M1 polarization. Blocking this process prevents pro-inflammatory activation, suggesting that macrophage polarization requires nuclear softening [95]. Interestingly, in murine macrophages, lamin A/C remains stable, but sun1/2, components of the LINC complex, are degraded, leading to reduced nuclear stiffness and enhanced polarization [96]. Preventing sun1/2 degradation increases nuclear stiffness and inhibits M1 activation. Sun1/2 knockout decreases nuclear stiffness, impairs phagocytosis, and limits chromatin accessibility at cytokine loci such as IL1b, indicating that structural remodeling of the nucleus initiates and sustains macrophage inflammatory responses [96]. Macrophages cultured on titania nanotube substrates exhibit reduced lamin A/C expression, disrupted actin polymerization, and impaired nuclear translocation of the mechanosensitive transcription coactivator MRTF-A, resulting in downregulation of pro-inflammatory genes such as IL6, TNFα, and IL1β [97]. In vivo, myeloid-specific deletion of lamin A/C leads to decreased LPS-induced expression of pro-inflammatory genes, including Il6, Tnf, and Ccl2, and protects against obesity-induced insulin resistance [98]. These studies demonstrate the critical role of nuclear mechanics in macrophage activation and polarization.

Dendritic cells lacking lamin A/C show reduced nuclear translocation of NF-κB after TLR stimulation and decreased chromatin accessibility at key proinflammatory and co-stimulatory gene loci [99]. These lamin A/C defective dendritic cells form fewer and smaller immunological synapses with naïve CD4^+^ T cells, failing to effectively prime T cell responses during viral infection [99]. Given lamin A/C’s known role in maintaining nuclear architecture, its absence likely alters nuclear mechanical properties, directly or indirectly impacting transcriptional responses to activation signals.

Besides lamins and nuclear envelope proteins, the 3D organization of chromatin, which also regulates nuclear stiffness (see section “How chromatin regulates nuclear mechanics”), is essential for orchestrating gene expression programs that underlie immune cell identity and function. In T cells, the 3D organization of chromatin drastically changes during naïve T cell activation, and these changes impact T cell differentiation into effector and memory T cells (for recent review, see [100]). For instance, chromatin is highly condensed in naïve T cells, and this condensed state is proposed to maintain the naïve T cell state [101]. T cell activation is accompanied by a global increase in chromatin accessibility and activation of key transcription factors such as STAT5 and AP-1 that remodel chromatin, suggesting that 3D chromatin reorganization is important for T cell activation [102]. Disrupting pathways involved in 3D chromatin organization, such as the SUV39H1-H3K9me3-HP1α pathway that regulates heterochromatin formation, impairs CD4+ T cells differentiation into T helper 2 cells, suggesting that 3D chromatin organization regulates T cell lineage stability [103]. Non-coding transcription directs enhancer-promoter communication, thereby establishing T cell identity [104] and lineage – specific transcription signature. Notably, disruptions in 3D genome architecture have been linked to immune dysregulation, contributing to autoimmune diseases, immunodeficiencies, and cancer [105,106]. Linking 3D chromatin organization, nuclear mechanics and T cell activation, a recent study showed that T cell activation induced by spreading on anti-CD3-coated glass, is accompanied by increased nuclear deformation along with accumulation and peripheral redistribution of repressive epigenetic marks, suggesting an increase in chromatin compaction during this type of T cell activation [107]. Interestingly, altering chromatin decompaction prior to activation impairs nuclear deformation and alters the immunological synapse’s morphology and molecular composition (actin accumulation and microtubule growth) but does not impair overall T cell activation [107]. Thus, this study indicates that 3D chromatin reorganization during T cell activation impacts the dynamics and molecular composition of the immunological synapse which might impair antigen extraction or cytotoxic function of T cells.

However, considering the intrinsic connection between nuclear architecture and gene expression [108], it is reasonable that altering the composition and structure of nuclear components, such as lamins and chromatin, impacts long-term immune cell pathways such as development and activation. It will be interesting to probe further the impact of nuclear mechanics on immune cells’ fate and potentially tease apart the genetic versus the biophysical role of the nucleus in immune cell development and activation (see section “Open questions and perspectives”).

### Nuclear mechanics regulate immune cell-mediated killing

Immune cells are professional killers with multiple mechanisms to kill other cells and pathogens. Arguably, the most drastic killing strategy employed by immune cells is extracellular trap release (ETosis). As we will discuss below, ETosis is a unique case of a non-genetic role of the nucleus in immune cell functions.

Upon detection of danger signals, various immune cells, including neutrophils, macrophages, mast cells, eosinophils, basophils, T cells and B cells, release chromatin to the extracellular space to form extracellular traps (ETs) in a process called ETosis [109–111]. Neutrophil extracellular traps (NETs) are composed of chromatin coated with antimicrobial and cytotoxic proteins/peptides, which can physically trap and neutralize pathogens and are thus important for immune response [110,112]. However, NETs are also released during sterile inflammation, can initiate the formation of microthrombi, kill host cells, damage organs and worsen the outcome of chronic inflammatory diseases, including cancer, diabetes and autoimmune diseases (for recent reviews, see [111,113–115]. NETosis can take two forms depending on its outcome: hour-long suicidal NETosis resulting in cell death [10,110,116,117] or minute-long vital NETosis after which neutrophils remain alive and can deploy more effector functions, such as migration and phagocytosis [118–120]. Importantly, while NETosis is accompanied by transcription firing [121], transcription is not required for suicidal NETosis [122] and the minute time-scale of vital NETosis is faster than gene expression. Thus, NETosis can be described as a transcription-independent process.

A hallmark of NETosis is chromatin decompaction as assessed by the increase in chromatin accessibility and the surface area occupied by chromatin [110,116,117,123,124]. Mechanistically, dissociation of histones from DNA is proposed to drive chromatin decompaction during NETosis. PAD4, an enzyme that converts arginine into citrulline, citrullinates histones and is important for efficient chromatin decompaction and NETosis [116,125]. Serine proteases, particularly neutrophil elastase, cleave histones during NETosis [10,126] and are important for NET release. Increasing histone acetylation also increases NETosis efficiency [127]. Thus, NETs are composed of decompacted chromatin, which adopts the ‘beads-on-string’ configuration of nucleosome-bound DNA. It is interesting to note that while nucleosomes are still found on NETs, nucleosomal histones are reported to be citrullinated by PAD4 or degraded by neutrophil elastase. Thus, it remains to be determined how citrullination and/or cleavage of histones drives chromatin decompaction during NETosis.

**Figure 3.**
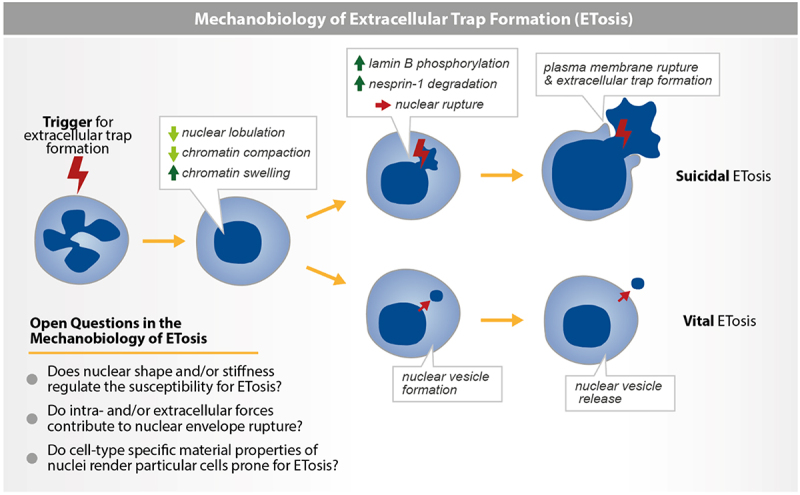
Mechanobiology of extracellular trap formation. Immune cells like neutrophils deploy extracellular traps (ETs) as a dramatic effector mechanism, commencing with a decrease in nuclear lobulation due to decreased chromatin compaction and increased swelling, eventually expelling chromatin and antimicrobial proteins to immobilize pathogens. Some cells survive this expulsion through vital ETosis, where vesicles bud from the nuclear envelope and are eventually released into the extracellular space, whereas others succumb to suicidal ETosis as their nuclei disintegrate entirely due to lamin B phosphorylation and nesprin-1 degradation. Subsequently, plasma membrane rupture leads to extracellular trap formation. Key questions persist: how do mechanical properties of the nucleus, such as shape and/or stiffness, dictate its ability to undergo ETosis? Do external forces dictate which cells undergo ETosis? Does chromatin swelling overpower the nuclear envelope and cause it to rupture?

Nonetheless, for NETs to trap and kill pathogens [110], shield cancer cells from other immune cells [128] or initiate thrombosis [113], chromatin must breach the nuclear envelope and reach the extracellular space. This requires cells to dynamically alter a critical mechanical feature of the nucleus – nuclear mechanical integrity. Two mechanisms have been proposed to drive the disruption of nuclear integrity during NETosis (Figure 3): 1) rupture of the nuclear envelope during suicidal NETosis; 2) vesiculation of the nuclear membrane during vital NETosis. Here, we will describe the limited but exciting data supporting the two mechanisms.

#### Disrupting nuclear integrity by nuclear envelope rupture

The nucleus drastically remodels before rupturing during both vital [118,119] and suicidal NETosis [116,117]. Several studies showed that neutrophils undergoing NETosis lose their stereotypical multilobulated nuclear shape [110,116,117,124]. Quantitative live microscopy of various types of neutrophils undergoing NETosis showed that the nucleus rounds up minutes after the onset of NETosis [116]. The rounding up of the nucleus occurs after vesiculation of the endoplasmic reticulum (ER) and depolymerization of actin, microtubules and vimentin intermediate filaments. Stabilizing the actin and microtubule network does not prevent nuclear rounding during NETosis suggesting that nuclear rounding is not the mere result of the relaxation of forces from the actin and microtubule cytoskeleton [116]. Whether vimentin disassembly or ER vesiculation participates in nuclear rounding during NETosis remains to be evaluated.

Independent of its mechanism, rounding of the nucleus during NETosis suggests that the mechanical properties of the nucleus are changing. Indeed, nuclear rounding decreases the nucleus’s surface-to-volume ratio, potentially increasing nuclear envelope tension [129–132]. We can also speculate that the vesiculation of the ER will reduce the excess membrane reservoir of the outer nuclear membrane, facilitating tension buildup at the nuclear membrane [131,133]. Direct measurement of nuclear envelope tension and its dependency on nuclear rounding will be essential to understand whether and how nuclear rounding during NETosis participates in the process.

Another drastic nuclear morphological change in suicidal NETosis is the rupture of the nuclear envelope. The lamina network, as well as the inner and outer nuclear membrane, rupture within minutes of each other during NETosis [10,116,124]. The lamina network has been shown to be phosphorylated during NETosis downstream of CDK6 activation [134] and/or PKC activation [135]. Li et al further showed that preventing lamin B phosphorylation prevents efficient NETosis in HL60-derived neutrophils [135]. A more recent report showed that, nesprin-1, another nuclear envelope protein, is degraded downstream of calpain 1 activation during NETosis [136]. From this data emerges a model where post-translational modification of nuclear envelope proteins (lamina, nesprin) leads to their dissociation from the nuclear envelope, rendering the nuclear envelope more fragile and prone to rupture. It remains to be determined whether this fragilized nuclear envelope ruptures spontaneously or through the action of mechanical forces. Further mechanical and structural characterization of the nuclear envelope during NETosis will be required to determine whether the posttranslational modification and dissociation of nuclear envelope proteins from the nucleus precede or follow the rupture of the nucleus.

An important area of research in NETosis is understanding whether and how chromatin decompaction can destabilize the nucleus and disrupt its mechanical integrity. Neubert et al. proposed that chromatin swelling and the resulting entropic elasticity mediate the plasma membrane rupture during NETosis [124]. Whether entropic elasticity similarly disrupts nuclear integrity remains unclear. Studies assessing the dynamics and impact of chromatin decompaction on the mechanical properties of the nucleus will be essential to determine whether and how chromatin decompaction dynamically alters the mechanical integrity of the nucleus during NETosis.

#### Disrupting nuclear integrity by nuclear membrane vesiculation

Following their first description of vital NETosis in 2007, Kubes et al. proposed that neutrophils undergoing vital NETosis release chromatin from the nucleus by vesiculating the nuclear membrane [137]. The authors showed by analysis of fixed time point electron micrographs that neutrophils stimulated for vital NETosis by exposure to *S. aureus* form dilated regions between the inner and outer nuclear membranes. These ‘nuclear blebs’ contained ‘beads-on-string’ polymers reminiscent of chromatin. Pilsczek et al. proposed that these nuclear blebs bud from the nucleus, leaving the rest of the nucleus intact, forming chromatin-containing vesicles that fuse with the plasma membrane to allow extracellular chromatin release [137]. This first stage of nuclear vesiculation was followed by the rupture/disintegration of the nuclear envelope, resulting in the formation of nucleus-free cells [137]. It will be exciting to elucidate the molecular and biophysical mechanisms of nuclear vesiculation during ETosis. Further, whether nuclear vesiculation alters the mechanical properties of the nucleus, leading to nuclear rupture/disintegration, remains to be determined.

More recently, Arya et al. reported that the nucleus vesiculates to release ETs without signs of nuclear disintegration during vital NETosis [120]. The authors showed that neutrophils migrating up an LTB4 chemokine gradient form LBR-positive vesicles containing histone H3, releasing up to three ~0.5 µm diameter vesicles in 1 hour while maintaining an intact-looking multilobulated nucleus. Mechanistically, the formation of these nuclear vesicles requires ceramide-rich membrane microdomains, LBR – proposed to mediate chromatin tethering to the NE-derived vesicles – and histone acetylation, thought to be mediated by LTB4 [120]. It remains to be determined how the formation of a limited number of nuclear vesicles (3 vesicles released in 1 hour) impacts the mechanical properties of the nucleus. Considering the requirement of ceramide-rich domains and LBR, a sterol reductase, further investigation on the role of nuclear lipids in forming these vesicles will advance our mechanistic understanding of how pathways that regulate lipid homeostasis impact the mechanical properties of the nucleus.

Whether the nucleus loses its integrity during NETosis through rupture, vesiculation, or a combination of both, NETosis offers a powerful framework to study how immune cells dynamically and systematically modulate nuclear mechanics to execute their host defense functions. Understanding this process may reveal the underlying principles that confer mechanical robustness to the nucleus. Moreover, NETosis provides a launching pad to explore whether other immune cells undergoing ETosis leverage shared or distinct mechanisms to disrupt nuclear integrity. Given the central role of nuclear breakdown in ETosis, several critical questions remain: i) how do cells physically breach nuclear integrity? ii) do the mechanical properties of the nucleus influence the efficiency of ETosis? iii) do mechanical forces from the surrounding microenvironment facilitate ETosis, and iv) does the unique nuclear architecture of immune cells predispose them to this form of cell death?

## What are the regulators of nuclear mechanics in immune cells?

Considering the critical role of the nucleus in immune cell function, the field needs to build a quantitative understanding of the regulators of nuclear mechanics. As stated earlier, the nucleus is a multilayered organelle, physically connected to the rest of the cell and the cytoskeleton by the LINC complex [4,138]. This allows mechanical force transmission and mechanical equilibrium between the nucleus and the rest of the cell. Nuclear mechanotransduction, which is the study of how forces are transmitted from the extracellular space, the cytoskeleton and the LINC complex to the nucleus, has been extensively reviewed elsewhere [4,138,139]. However, while we have a growing list of the nuclear proteins/structures that allow the nucleus to sense and respond to mechanical forces, we have a blurry picture of which nuclear components regulate the mechanical property of the nucleus – thus the ability of the nucleus to withstand or push against mechanical forces. Here, we will discuss our current knowledge of the intranuclear regulators of nuclear mechanics in immune cells. We will also draw inspiration from nonimmune cells to work toward building an intuition of what regulates the mechanical properties of the nucleus in immune cells.

### The nuclear envelope as a protective elastic shell

The nuclear envelope, which consists of the double lipid bilayer of the nuclear membrane, the nuclear pore complex (NPCs) and the nuclear lamina, provides a physical interface between the nucleus and the rest of the cell. The most common mechanical model of the nucleus is an elastic shell (i.e., nuclear envelope) enclosing a viscoelastic material (i.e., the nucleoplasm) [62,140,141].

The nuclear lamina, composed of A and B-types of lamins intermediate filaments, is proposed to set the stiffness of the nuclear envelope. From a physical standpoint, stiffness is a measure of how much energy a material will require to deform. Seminal work by Lammerding et al. demonstrated that the downregulation of lamin A/C in fibroblasts decreases nuclear stiffness as measured by increased nuclear deformation upon cell stretching [89]. For the past 20 years, multiple studies have further established the critical role of lamins (both A and B-type lamins) in regulating nuclear stiffness/elastic response but also viscosity [142–144]. The current model in the field – primarily based on studies from nonimmune cells – is that lamins regulate both the short-term/elastic and long-term/viscous mechanical responses of the nucleus to large deformations (>30% strain) [145], as well as the strain-stiffening behavior of the nucleus [145–147]. It is proposed that the ratio between lamin A/C and lamin B (lamin A:B), rather than the total amount of lamin A/C, regulates nuclear stiffness and deformability: the higher the lamin A:B ratio, the stiffer the nucleus [38,59].

**Figure 4.**
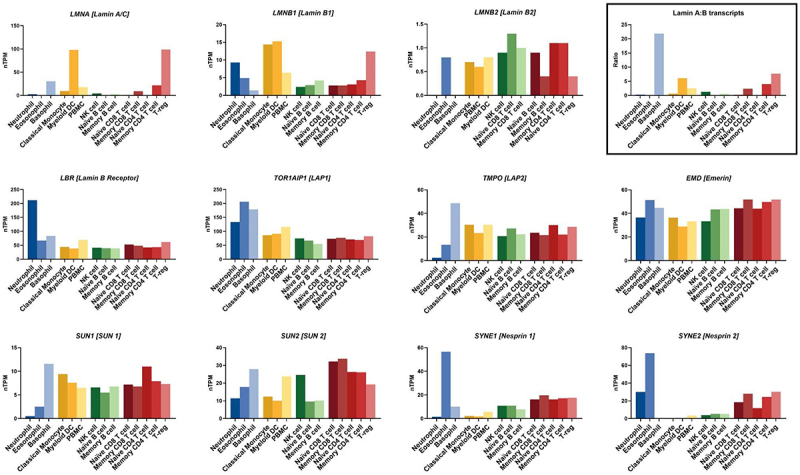
Immune cells differentially express nuclear envelope proteins important for nuclear mechanics and mechanotransduction. Normalized transcript expression (nTPM, transcripts per million) of genes encoding components involved in nuclear structure and mechanotransduction, shown across various immune cell populations. Data from the human protein atlas, www.proteinatlas.org, (uhlén M et al, 2015). Genes include LMNA, LMNB1, LMNB2, LBR, TOR1AIP1, TMPO, EMD, SUN1, SUN2, SYNE1 and SYNE2. Inset shows the ratio of LMNA to the sum of LMNB1 and LMNB2 transcripts.

Immune cells dynamically express various levels of lamin proteins as they differentiate and get activated upon detecting danger signals [148–152] (Figure 4). CD34+ hematopoietic stem cells (CD34+ HSCs), which give rise to erythroblast, megakaryocytes, lymphoid and myeloid cells, have a low lamin A:B ratio of 0.2 and nuclei with a stiffness of 7.04 ± 4.07 kPa [59,153]. Late erythroblasts that give rise to enucleated red blood cells have a high lamin A:B ratio of 11 (100x more than CD34+ HSCs) and an associated nuclear stiffness of ~5–6 kPa that increases with increasing lamin A:B ratio through knockdown of lamin B1. Megakaryocytes which give rise to platelets, upregulate both A- and B-types lamins up to 10-fold during maturation but maintain an intermediate level of lamin A:B ratio of 2–3 which associate with a nuclear stiffness of ~200 Pa. T cells, which have a lamin A:B ratio of 0.7, have a nuclear stiffness of ~25 Pa. T cells upregulate lamin A during activation which results in increased nuclear stiffness in activated T cells [92]. Neutrophils downregulate lamin A/C and B1 and upregulate lamin B2 expression during differentiation [154,155]. While we lack quantitative measurements of the lamin A:B ratio and stiffness of neutrophils’ nuclei, the lower expression level of lamin A/C in neutrophils has been proposed to increase nuclear lobulation and deformability through micron-sized pores [51]. Downregulation of lamin A/C in immature dendritic cells relieves the need for the perinuclear actin-mediated nuclear compression during migration through micron-sized pores [9], suggesting that the amount of lamin A/C correlates with nuclear stiffness in immature dendritic cells. From this limited yet impactful body of literature emerges a model where, comparable to nonimmune cells, immune cell nuclear stiffness scales with the amount of lamins and potentially the lamin A:B ratio. It is interesting to note that the immune cell progenitor CD34+, which has a low lamin A:B ratio and a stiff nucleus, breaks this correlation, indicating that other nuclear envelope proteins regulate nuclear mechanics.

In addition to lamins, multiple other nuclear envelope proteins regulate nuclear envelope structure and mechanical properties in nonimmune cells. For example, LAP1C, which connects the inner nuclear membrane with the nuclear lamina, supports cell migration through constrained environments, suggesting LAP1C’s importance in nuclear deformability [156]. LAP2β, which resides in the inner nuclear membrane, prevents nuclear rupture upon stretching of lamin B1-deficient cells, implying that LAP2β enhances nuclear mechanical stability [157]. Emerin and MAN1, which primarily reside in the inner nuclear membrane, have also been shown to increase nuclear envelope stability [157]. LINC complex proteins like nesprins and sun1/2, which connect the nuclear envelope to the cytoskeleton and are downregulated in neutrophils [158], allow the transmission of mechanical forces between the nucleus and the rest of the cell [159,160]. While the roles of these proteins in force transmission to the nucleus are well established [160], we have a limited quantitative understanding of whether these proteins regulate the mechanical properties (e.g., stiffness, deformability) of the nucleus in immune cells. Further studies are needed to understand how the ensemble of proteins that reside in the nuclear envelope regulates the mechanical properties and stability of the nucleus.

Two other major structural elements of the nuclear envelope are the nuclear membrane and the nuclear pore complex. Although these elements have been less studied than the nuclear lamina, it is important to discuss their potential roles in regulating the mechanical properties of the nucleus in immune cells.

### How the nuclear membrane regulates nuclear mechanics

**Figure 5.**
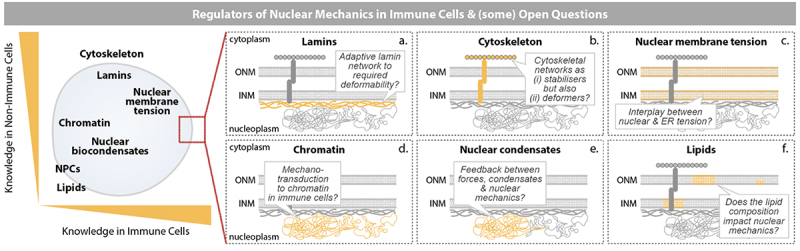
Regulators of the mechanobiology of immune cell nuclei. (a) Nuclear lamins, a class of intermediate filament proteins, form a dense meshwork beneath the inner nuclear membrane and play a central role in defining the mechanical behavior of the nucleus over time. They contribute to its elastic, viscous, and viscoelastic properties, governing how the nucleus responds to mechanical stress. Importantly, the abundance and composition of lamins particularly the lamin A to lamin B ratio, directly influence nuclear stiffness and deformability, enabling the nucleus to adapt to different cellular environments. (b) The cytoskeleton plays a crucial and dynamic role in nuclear mechanics, acting both as a mechanical support system and as a bridge for force transmission. Its components are intimately connected to the nucleus via the LINC complex and contribute to the regulation of nuclear shape, positioning, deformation, and response to mechanical stress. (c) Deformations of the nucleus act onto its outer (ONM) and inner nuclear membrane (INM), causing the unfolding of nuclear membrane wrinkles and alterations in nuclear membrane tension. (d) Chromatin, along with its tethering to the nuclear lamina and its compartmentalization into functional subdomains, influences nuclear mechanics through its intrinsic polymeric properties, contributing to nuclear stiffness. Moreover, mechanical stimuli can dynamically modulate genome organization, promoting active or repressive transcriptional states that ultimately affect cell behavior and survival. (e) Nuclear condensates contribute to the spatial organization of chromatin and regulation of gene expression by serving as dynamic molecular hubs. Recent evidence suggests they regulate chromatin structure and stiffness, and participate in nuclear mechanosensing. (f) Lastly, the lipid composition of the nuclear membrane (cholesterol, triglycerides) has been proposed to affect the nuclear mechanical properties.

The nuclear membrane is the first interface between the nucleus and the rest of the cell. It is constituted by a double lipid bilayer connected by the nuclear pore complex (NPCs). The lipid bilayer physically separates the nuclear content from the cytoplasm, while NPCs regulate the transport of material between them (recently reviewed in [161]). The common view is that by analogy to the plasma membrane, the lipid bilayer has a limited impact on the mechanical properties of the nucleus. This is based on the fluid-mosaic model of the membrane, which states that, similar to fluids, membranes flow under force and provide minimal mechanical resistance to deformation [162–164]. However, recent research indicates that tension can build in the nuclear membrane [6,53,165], suggesting that the nuclear membrane (Figure 5) can provide mechanical resistance to the nucleus and participate in nuclear mechanobiology.

How is this achieved? Three key factors are unique to the nuclear membrane: 1) it is a double lipid bilayer whose bending modulus can be 10-fold higher than for a single bilayer [166] 2) it is continuous with the large reservoir of membranes that constitutes the endoplasmic reticulum. This raises the question of how tension can build at the nuclear membrane; 3) it contains a dense array of NPCs known to change their structures upon mechanical forces. It will be exciting for the field to understand the emerging properties of such a composite material. Here, we will discuss our limited knowledge of how nuclear lipids and NPCs (might) regulate nuclear mechanics.

#### Lipids in nuclear mechanics

Immune cells provide a unique opportunity to understand how lipid composition impacts nuclear mechanics (Figure 5). For instance, the multilobulated shape of neutrophils’ nuclei correlates with increased expression of LBR. Neutrophils from patients with decreased or mutated LBR have less segmented/round nuclei, which correlate with diseases such as the mild Pelger-Huët anomaly and the lethal Greenberg skeletal dysplasia [167,168]. These disease-related mutations in LBR alter the sterol reductase activity of the protein and correlate with altered cholesterol composition and nuclear mechanics in other cells [167,168]. This suggests that the lipid composition of the nucleus (for instance, the amount of cholesterol) regulates the mechanical properties of the nucleus.

Interestingly, neutrophils have a unique expression level of major lipid remodeling enzymes [169]. Besides LBR, neutrophils highly express DGAT2 [170] and cyclooxygenase-2 (COX-2) (human protein atlas [171]), which regulate the biosynthesis of triglycerides and prostaglandins, respectively [172,173]. Neutrophils downregulate lipin 1 (human protein atlas [171]), a phosphatidic acid phosphatase that regulates triglyceride and glycerophospholipid metabolism [174]. Interestingly, all these enzymes are reported to be localized in the nucleus [175]. We have a poor understanding of whether and how these lipid remodeling enzymes regulate the mechanical properties of the nucleus. Studies evaluating the impact of lipid metabolism on nuclear mechanobiology will be important to broaden our understanding of how the nuclear membrane regulates nuclear mechanics and downstream functions of neutrophils or other immune cells.

Nevertheless, borrowing concepts from studies of how lipid composition impacts the mechanical properties of giant unilamellar vesicles (GUVs) can help us speculate on how the lipid composition of the immune cell nuclei could impact nuclear mechanics. For instance, increasing the amount of acidic lipids such as phosphatidic acid (PA; negatively charged; precursor of other phospholipids) or phosphatidylglycerol (PG; negatively charged; 1–2% of mammalian cell lipids) decreases the deformability of phosphatidylcholine (PC; neutral; ~33% of phospholipids in mammalian cells) or phosphatidylethanolamine (PE; weakly acidic; ~20% of phospholipids in mammalian cells) containing GUV [176–178]. Increasing the amount of cholesterol (which represents up to 50% of plasma membrane lipids) increases the deformability of sphingomyelin-containing GUVs [179] but decreases the deformability of GUVs containing saturated and mono-saturated PC lipids [180–182]. Finally, increasing lipid saturation decreases the deformability of GUVs containing lipids derived from yeast [183]. While we have yet to build a clear picture of the lipid composition of immune cells’ nuclei, it is tempting to speculate that the increased LBR expression in neutrophils might increase the amount of cholesterol at the nuclear membrane. How this might impact nuclear deformability will depend on the amount of sphingolipids and saturated lipids at the nuclear membrane. Combining recent advances in quantitative imaging of lipids in mammalian cells [178] and measurement of nuclear mechanics (see Table 1) will allow us to map the dynamics of nuclear lipids and probe how these lipids regulate nuclear mechanics and downstream immune cell functions.

#### Nuclear pore complexes in nuclear mechanics

Nuclear pore complexes (NPCs) are large macromolecular structures mainly studied for their role in regulating nucleo-cytoplasmic transport. Research from recent years indicates that mechanical forces on the nucleus induce NPCs to stretch, change in structure and alter their permeability range, indicating that NPCs participate in nuclear mechanotransduction [184–186].

Although several NPC proteins were demonstrated to play critical roles in immune cell functions [187], and immune cells experience large forces which result in large nuclear deformation (for instance, during confined migration), we have a limited understanding of whether force-induced changes in NPC structure or permeability impact the mechanical properties of the nucleus and downstream immune cell functions. Hypothetically, changes in NPC permeability will change the number of macromolecules that can flow through the nuclear membrane. This could impact nuclear volume, crowding and macromolecular osmotic pressure, all of which could change the mechanical properties and functions of the nucleus in immune cells. Studies testing this hypothesis will advance our understanding of the impact of NPC structure on immune cell nuclear mechanics and mechanotransduction.

### How chromatin and other nucleoplasmic structures regulate nuclear mechanics

#### How chromatin regulates nuclear mechanics

Chromatin allows cells to pack and organize their meter-long DNA into the micron-sized nucleus. Chromatin is organized into loosely and densely packed regions [188]. The loosely packed/decompacted regions of chromatin are associated with specific epigenetic marks (e.g., histone acetylation such as H3K27Ac [189]), which are associated with transcription activation: these regions are commonly called euchromatin [190]. The densely packed/compacted regions of chromatin are associated with specific epigenetic marks (e.g., histone methylation such as H3K9me3, H3K27me3) [191] which are ‘read’ by specific proteins (e.g., HP1α, Polycomb) [192] and are associated with transcription repression: these regions are commonly called heterochromatin and tend to localize at the nuclear periphery and specific subnuclear structures (nucleoli, nuclear speckle, polycomb bodies) [193]. This intricate physical and biochemical organization of chromatin in the nucleus allows cells to control gene expression dynamically [194]. As such, the impact of chromatin organization on cell function has been mainly studied in the context of gene expression.

In nonimmune cells, changes in chromatin state (compaction, organization, etc.) have been shown to regulate nuclear structure and deformability. For instance, chromatin decompaction via an increase of histone acetylation decreases nuclear stiffness [145], promotes nuclear blebbing [195], and facilitates an auxetic response to pressure [196]. Chromatin compaction (increase in heterochromatin) stiffens the nucleus as measured by decreased nuclear creep [141] or response to small deformation [145]. Rapid degradation of HP1α, a reader of H3K9me3 that crosslinks chromatin in heterochromatin, decreases nuclear stiffness and circularity [197]. Force application on the nucleus decreases epigenetic marks associated with heterochromatin (H3K9me) and correlates with a decrease in nuclear stiffness and membrane tension/lipid order [198]. This mechanically induced heterochromatin loss is proposed to insulate the genome against DNA damage and mechanical force [198]. Disconnecting chromatin from the nuclear membrane in *S. pombe* yeast cells that lack lamins decreases the stiffness and viscosity of the nucleus, supporting the model that tethering chromatin at the nuclear periphery is important for the viscoelastic response of the nucleus [199]. Computational and polymer physics-based modeling showed that nuclei without chromatin shrink and buckle upon stretch. Interestingly, this study shows that the amount of heterochromatin and the level of heterochromatin–heterochromatin crosslink regulate nuclear stiffness but only if heterochromatin is tethered to the nuclear lamina. The model used in that study predicts that the heterochromatin-lamina domains expand upon nuclear deformation, suggesting that chromatin-lamina tethering allows external forces to be transmitted to the overall chromatin network in mammalian cells [200]. From these works emerges the model that heterochromatin, and potentially its tethering to the nuclear periphery, increases nuclear stiffness and viscosity.

In immune cells, perhaps the most evident impact of chromatin compaction state on nuclear mechanics is observed during NETosis. As stated earlier, the drastic alteration of chromatin organization during NETosis associates with a change in nuclear shape and mechanical stability [116,135]. Current evidence suggests that chromatin decompaction might be necessary for nuclear rupture during NETosis [10,124]. Since NETosis is a transcription-independent process [122], it allows us to probe the biophysical role of chromatin without the added genetic effects of chromatin. Thus, systematic characterization of nuclear mechanics as a function of chromatin reorganization during NETosis will allow us to mechanistically understand whether and how chromatin organization regulates the mechanical properties of the nucleus in immune cells.

Beyond NETosis, we have a limited understanding of how the compaction state of chromatin responds to force and impacts nuclear mechanics in immune cells. It has recently been proposed that after confined migration through 5 µm pores, neutrophils can alter their heterochromatin to protect their transcriptionally active sites on chromatin [201]. In myeloid progenitor cells, it has been observed that disruption of loop-extrusion factors via acute depletion of nipped-B-like protein (NIPBL) changes the accessibility landscape of chromatin but also impacts nuclear shape, since NIPBL-depleted progenitors adopted polymorphonuclear shapes [202]. T cells adhesion on VCAM1, an integrin ligand critical for lymphocytes extravasation and activation [203], increases H3K9 methylation, chromatin compaction, nuclear stiffness and T cell migration through collagen gels and narrow transwell pores, independently of transcription [204]. Interestingly, disruption of G9a, the enzyme that methylate H3K9 in this condition, impairs T cell migration in collagen gels suggesting that nuclear stiffening through chromatin compaction facilitates cell deformation through narrow pores in T cells [204]. While this study challenges the classical view that nuclear stiffening impairs migration through narrow pores, it corroborates the crucial role of chromatin in affecting nuclear morphology, structure and mechanics.

Chromatin architectural proteins, histones, and their PTMs are well-established regulators of nuclear structure and chromatin compaction [205]. Recently, an additional layer of complexity has been identified within nuclear organizing structures, suggesting the existence of further mechanisms contributing to the mechanical regulation of the nucleus. This emerging concept will be further explored in the following paragraph.

#### How nuclear condensates regulate nuclear mechanics

Complementing the role of structural proteins in shaping chromatin architecture, biomolecular condensates have emerged as key regulators of nuclear biophysical properties, influencing chromatin organization, gene expression, and nuclear mechanics (Figure 5). From a biophysical perspective, two main phase separation mechanisms have been proposed to explain the formation of nuclear condensates: polymer–polymer phase separation (PPPS) and liquid–liquid phase separation (LLPS) [206–208]. In PPPS, chromatin-binding proteins mediate bridging interactions with the DNA scaffold, promoting the compaction of chromatin into ordered globular structures. In contrast, during LLPS, chromatin-associated proteins engage in multivalent interactions among themselves, forming liquid-like protein droplets that can persist independently of the chromatin scaffold [207]. These phase-transition processes establish membrane-less boundaries, enabling the spatial segregation and optimization of diverse activities within the complex nuclear environment [209].

Phase-separated chromatin bodies are associated with both heterochromatic domains – characterized by high chromatin density and transcriptionally silent genomic regions – and euchromatic domains, where gene expression is regulated by the condensation of the transcriptional machinery at enhancer-rich gene clusters [208,210]. In addition to their genetic functions, chromatin bodies may influence nuclear architecture and rigidity by exerting mechanical forces that shape genome organization [211–213].

Although most foundational work in this field has focused on nonimmune cell models, several lines of evidence suggest that biomolecular condensates contribute to nuclear mechanical properties, offering potential insights into the mechanobiology of the immune system. For example, the heterochromatin protein HP1α, which undergoes phase separation to maintain heterochromatin integrity [208], has been shown to provide mechanical strength to chromosomes and the nucleus throughout the cell cycle [197]. Furthermore, HP1α degradation leads to decreased chromatin stiffness without altering transcription or histone methylation, pointing to a mechanistic decoupling of its structural and regulatory functions [197]. Additionally, an imbalance between active and repressive chromatin condensates, as observed in Kabuki syndrome, results in altered nuclear mechanical properties, with important implications for disease pathogenesis [214], suggesting an important nexus between chromatin bodies and cell mechanical functions. Further evidence suggests that nuclear condensates may be mechanosensitive, capable of responding to external mechanical stimuli [215–219]. For instance, migration of nonimmune cells through spatially restricted environments imposes mechanical strain on the chromatin architecture, promoting the deformation and subsequent coalescence of intranuclear biomolecular condensates, including nucleoli and nuclear speckles [217]. These observations demonstrate that mechanical perturbations of chromatin architecture influence the assembly and biophysical characteristics of nuclear condensates, potentially playing a role in cellular mechanosensing and mechanotransduction.

The current understanding of nuclear biomolecular condensates in the immune system primarily centers on their roles in establishing and maintaining immune cell identity and fate [220]. Importantly, condensate dysregulation has been linked to disrupted immune cell differentiation, aging and cancer [221–225]. Notably, transcriptional condensates were shown to be essential for B cell differentiation [221]. Specifically, the intrinsically disordered domain (IDD) of the transcription factor EBF1 facilitates the opening of closed chromatin regions at B cell fate-instructive loci [221]. Moreover, the IDD of the transcription factor TCF-1 is required to drive T cell lineage commitment [223]. Another study reports that a mutant variant of the nucleolar protein NPM1, frequently found in acute myeloid leukemia, associates with specific chromatin regions and gives rise to aberrant transcriptional condensates that sustain the expression of leukemia-promoting genes [225].

While additional studies are required to unravel the molecular mechanisms underlying condensate formation and their coupling to force generation and mechanosensing, it is plausible that chromatin bodies play essential roles in regulating immune cell mechanics and functional outputs. Notably, their assembly in response to mechanical cues may contribute to nuclear structural integrity, mitigating nuclear envelope rupture during migration through confined environments, or alternatively, act as mechanotransductive hubs that initiate gene expression programs critical for cell migration and other immune cell functions.

## Open questions and perspectives

The past 20 years have transformed our understanding of how immune cells regulate the mechanical properties of the nucleus and how these properties in turn regulate immune cell functions, ranging from development, trafficking, to pathogen killing and the propagation of unwanted inflammation. As the field of nuclear mechanobiology in immune cells grows, we are excited for all the new knowledge and potential applications that will be generated. Below, we briefly discuss some open questions that will nucleate further interest in understanding the mechanical role of the nucleus in immune responses.

### Build a quantitative understanding of the origin of nuclear deformability

Research on immune cells’ rapid and efficient migration strategies has taught us fundamental principles of how cells solve the riddle of moving through space-restricted tissues while carrying the nucleus as a bulky physical cargo. However, while it is intuitively accessible to understand that nuclear deformability is essential for migration through narrow environments, we still have a limited understanding of the specific mechanical properties of the nucleus that regulate its deformability. Is the nuclear volume, shape, stiffness, surface tension, or a combination of these properties the key parameter that dictates its deformability? Solving this question will require directly measuring these mechanical properties across all immune cell types in vitro, but also in their native tissue environment. Such an experimental approach could be complemented with a theoretical or computational framework to define the overarching principles of nuclear mechanics. Thus, a systematic characterization of the mechanical properties of immune cells at a steady state is a crucial step toward understanding the design principle of the nucleus in immune cells and beyond.

### Investigate how nuclear-cytoskeleton force transmission occurs in immune cells

Understanding the mechanobiology of immune cell nuclei will require further characterization of how the nucleus is coupled to the cytoskeleton. This characterization would benefit from studies on cytoskeleton networks independent of actomyosin. Furthermore, studying immune cells such as neutrophils, which are devoid of a classical LINC complex [158], might reveal alternative force transmission mechanisms between the nucleus and the cell. Considering that compressive forces from restrictive environments can be directly transmitted to the nucleus, independently of classical force-sensing mechanisms present on the cell surface [8,9], it will be important to investigate the interdependence of force-sensing mechanisms and their reciprocal feedback at different cellular levels (e.g., cell surface, cytoskeleton, and organelles like the nucleus). Finally, understanding how cells integrate signals from nuclear mechanobiology, such as nuclear gauging and space-sensing, with signals from chemokine receptors, cytoskeleton dynamics, and other organelles and their mechanics, will enable the discovery of the principles in cellular decision-making.

### Tease apart the mechanical from the genetic functions of nuclear envelope proteins in nuclear mechanobiology

Research on how nuclear mechanics regulates immune cell differentiation, migration, and effector functions yielded exciting discoveries, primarily on the role of lamins and other nuclear envelope proteins. However, as lamins impact not only nuclear mechanics but also gene expression, it remains unclear whether perturbation of nuclear mechanics versus gene expression regulates immune cell fate and functions. Furthermore, as hundreds of proteins constitute the nuclear envelope [175], does one of these proteins regulate nuclear mechanics independently of gene expression? Thus, further mechanistic studies will be required to understand whether and how alteration of nuclear envelope composition and structure directly impact nuclear mechanics and downstream immune cell fate and functions independently of second messengers controlled by gene expression.

### Build an understanding of how chromatin regulates nuclear mechanobiology in immune cells

Chromatin is emerging as a major regulator of nuclear mechanobiology. The current model, primarily derived from research in nonimmune cells, proposes that as a stiffer polymer, heterochromatin increases nuclear stiffness at small deformation. How this is achieved remains an open question. Is this through blocking the transcription of genes that directly regulate nuclear structure and mechanics? Is this through the regulation of bulk nuclear stiffness? Or through an increase in effective nuclear surface tension, considering the connection between heterochromatin, the nuclear lamina, and the nuclear membrane? The compaction state of chromatin has also been proposed to regulate nuclear and cell volume. Is chromatin regulating nuclear mechanics through the regulation of nuclear crowding, osmotic pressure, or volume? These are important questions for the field to be addressed to build a quantitative picture of how chromatin regulates nuclear mechanics. Given the transcriptional independence and fast timescales of immune cell processes such as migration and NETosis, immune cells provide a unique model to dissect how chromatin regulates the mechanical properties and function of the nucleus independently of gene expression.

### Further assess the in vivo impact of nuclear mechanobiology in immune cells

Overall, studying the spatiotemporal dynamics of nuclear mechanobiology in mammalian immune cells in vivo has been particularly challenging due to their short lifespan, rapid migration, and lack of proliferation. As a result, microfabrication has become a standard approach in the field, allowing researchers to investigate the biophysical dynamics of confined immune cell migration in vitro by providing spatial control through defined geometries, confinement degrees, and cellular paths. However, notably, studies in Drosophila, fish and mouse ear explants suggest that the concepts identified in vitro are required for in vivo immune cell functions. Further, several human diseases correlate with mutations in nuclear genes regulating nuclear mechanobiology. These diseases include laminopathies [226], premature aging [227], and cancer [228], which are also characterized by immune cell dysfunction [229–231]. Thus, understanding how dysregulated nuclear mechanobiology in immune cells alters immune cell fate and function might provide a new mechanistic understanding of the emergence of these diseases. In the future, it will be important to establish additional in vivo and ex vivo models to investigate the mechanobiology of immune cell nuclei in native healthy and diseased tissue environments, such as by building on the blooming fields of organs-on-chip or 3D bio-printed organs [232].

### Leverage immune cells to advance our understanding of nuclear mechanobiology

The mechanical properties and function of the nucleus actively regulate immune cell fate and function. Therefore, understanding how immune cells regulate the mechanical properties of the nucleus and how these properties, in turn, influence immune cell functions, is essential for advancing our knowledge of immune responses. Beyond immunity, the distinctive mechanical features of immune cell nuclei offer a powerful model system to deepen our understanding of nuclear mechanobiology more broadly. For instance, the low expression level of lamin A/C in many immune cells can allow us to understand how other nuclear components regulate nuclear mechanobiology. The neutrophil’s ability to migrate through 1 µm^2^-sized pores and survive such transient but extreme deformations, enables us to identify the limits of nuclear deformability and integrity, and whether they contribute to the overall short lifespan of many immune cells. NETosis allows us to understand how nuclear mechanics remain dynamic without the cytoskeleton. The fact that NETosis is transcription-independent can help us understand how nuclear mechanics are regulated independently of transcription and allow us to probe deeper into the non-genetic role of chromatin and the nucleus. Thus, the unique properties and functions of immune cells provide a unique opportunity to test existing concepts of nuclear mechanobiology in highly dynamic cells and to discover novel fundamental concepts in nuclear mechanobiology.

### Learn from nuclear mechanobiology to understand organelle mechanobiology

Although the nucleus has distinct physical properties (shape, size, structure …) when compared to other organelles, the tools developed and insights gained from studying nuclear mechanobiology may provide general frameworks for studying the less-explored fields of how other organelles, including membrane-surrounded organelles like mitochondria and non-membrane-surrounded organelles like the centrosome, sense, withstand, and respond to mechanical forces. In addition, understanding how a large and stiff organelle like the nucleus impacts the mechanical properties and functions of immune cells may also teach us about the mechanobiology of disease-relevant foreign intracellular cargos, such as microplastics [233] and intracellular parasites like Toxoplasma gondii [234]. Thus, studying the physical principles and the molecular and cellular mechanisms of nuclear mechanobiology has broad implications for immune cell biology and pathophysiology.

### Harness nuclear mechanobiology for therapeutic purposes

As our understanding of the impact of nuclear mechanobiology in immune cells grows, it opens the exciting possibility of designing strategies to control nuclear mechanics for therapeutic purposes. For instance, could we enhance CAR T cell penetration in solid tumors and downstream cancer cell killing by increasing the deformability of CAR T cell’s nucleus? Conversely, could we prevent unwanted neutrophil recruitment to inflamed tissues of patients with chronic inflammatory diseases by decreasing the deformability of the neutrophil’s nucleus? How could we achieve this in a specific yet minimally invasive way? While genetic engineering allows us to specifically alter the composition and mechanical properties of CAR T cell’s nucleus, the long-time scale of this approach makes it challenging to apply to other immune cells such as the short-lived neutrophils. Small molecules that modulate the assembly, amount or activity of nuclear proteins such as lamins might allow us to control the mechanical properties of the nucleus in all cells but targeting these molecules to specific immune cells in vivo might be challenging. Thus, translating our understanding of nuclear mechanobiology to control immune cell functions for improved health will provide the opportunity to develop new therapeutic approaches.

## Data Availability

No Data were generated in this work. Data in Figure 4 were obtained from the Human protein Atlas (www.proteinatlas.org, (Uhlén M et al, 2015)) and are available at the following URL: v22.proteinatlas.org/humancell

## References

[1] Bruusgaard JC, Liestøl K, Ekmark M, et al. Number and spatial distribution of nuclei in the muscle fibres of normal mice studied in vivo. J Physiol. 2003;551(2):467–36. doi: 10.1113/jphysiol.2003.045328 12813146 PMC2343230

[2] Ulloa R, Corrales O, Cabrera-Reyes F, et al. B cells adapt their nuclear morphology to organize the immune synapse and facilitate antigen extraction. Front Immunol. 2022;12:801164. doi: 10.3389/fimmu.2021.801164 35222354 PMC8863768

[3] Bustin M, Misteli T. Nongenetic functions of the genome. Science. 2016;352(6286):aad6933. doi: 10.1126/science.aad6933 27151873 PMC6312727

[4] Kalukula Y, Stephens AD, Lammerding J, et al. Mechanics and functional consequences of nuclear deformations. Nat Rev Mol Cell Biol. 2022;23(9):583–602. doi: 10.1038/s41580-022-00480-z 35513718 PMC9902167

[5] Kirby TJ, Lammerding J. Emerging views of the nucleus as a cellular mechanosensor. Nat Cell Biol. 2018;20(4):373–381. doi: 10.1038/s41556-018-0038-y 29467443 PMC6440800

[6] Lomakin AJ, Cattin CJ, Cuvelier D, et al. The nucleus acts as a ruler tailoring cell responses to spatial constraints. Science. 2020;370(6514):eaba2894. doi: 10.1126/science.aba2894 33060332 PMC8059074

[7] Alraies Z, Rivera CA, Delgado M-G, et al. Cell shape sensing licenses dendritic cells for homeostatic migration to lymph nodes. Nat Immunol. 2024;25(7):1193–1206. doi: 10.1038/s41590-024-01856-3 38834865 PMC11224020

[8] Renkawitz J, Kopf A, Stopp J, et al. Nuclear positioning facilitates amoeboid migration along the path of least resistance. Nature. 2019;568(7753):546–550. doi: 10.1038/s41586-019-1087-5 30944468 PMC7217284

[9] Thiam H-R, Vargas P, Carpi N, et al. Perinuclear Arp2/3-driven actin polymerization enables nuclear deformation to facilitate cell migration through complex environments. Nat Commun. 2016;7(1):10997. doi: 10.1038/ncomms10997 26975831 PMC4796365

[10] Thiam HR, Wong SL, Wagner DD, et al. Cellular mechanisms of NETosis. Annu Rev Cell Dev Biol. 2020;36(1):191–218. doi: 10.1146/annurev-cellbio-020520-111016 32663035 PMC8499668

[11] Janeway CA Jr, Travers P, Walport M, et al. Chapter 9, The humoral immune response. In: Immunobiology: the immune system in health and disease. 5th ed. New York: Garland Science; 2001. Available from: https://www.ncbi.nlm.nih.gov/books/NBK10752/

[12] Janeway CA Jr, Travers P, Walport M, et al. Immunobiology: the immune system in health and disease. In: Principles of innate and adaptive immunity. 5th ed. New York: Garland Science; 2001. Available from: https://www.ncbi.nlm.nih.gov/books/NBK27090/

[13] Xu H, Li Y, Gao Y. The role of immune cells settled in the bone marrow on adult hematopoietic stem cells. Cell Mol Life Sci. 2024;81(1):420. doi: 10.1007/s00018-024-05445-3 39367881 PMC11456083

[14] Farber DL. Tissues, not blood, are where immune cells function. Nature. 2021;593(7860):506–509. doi: 10.1038/d41586-021-01396-y 34035530

[15] Grönloh MLB, van der Meer WJ, Tebbens ME, et al. Leukocyte transendothelial migration hotspots at a glance. J Cell Sci. 2025;Jun 1;138(11):jcs263862. doi:10.1242/jcs.263862 40527297

[16] Kameritsch P, Renkawitz J. Principles of leukocyte migration strategies. Trends Cell Biol. 2020;30(10):818–832. doi: 10.1016/j.tcb.2020.06.007 32690238

[17] Nourshargh S, Alon R. Leukocyte migration into inflamed tissues. Immunity. 2014;41(5):694–707. doi: 10.1016/j.immuni.2014.10.008 25517612

[18] Silver FH, Christiansen DL. Mechanical properties of tissues. In: Biomaterials science and biocompatibility. New York, NY: Springer. 1999. doi: 10.1007/978-1-4612-0557-9_7

[19] Bousso P. T-cell activation by dendritic cells in the lymph node: lessons from the movies. Nat Rev Immunol. 2008;8(9):675–684. doi: 10.1038/nri2379 19172690

[20] Förster R, Braun A, Worbs T. Lymph node homing of T cells and dendritic cells via afferent lymphatics. Trends Immunol. 2012;33(6):271–280. doi: 10.1016/j.it.2012.02.007 22459312

[21] Grant SM, Lou M, Yao L, et al. The lymph node at a glance – how spatial organization optimizes the immune response. J Cell Sci. 2020;133(5):jcs241828. doi: 10.1242/jcs.241828 32144196 PMC7063836

[22] Du H, Bartleson JM, Butenko S, et al. Tuning immunity through tissue mechanotransduction. Nat Rev Immunol. 2023;23(3):174–188. doi: 10.1038/s41577-022-00761-w 35974148 PMC9379893

[23] Sutherland TE, Dyer DP, Allen JE. The extracellular matrix and the immune system: a mutually dependent relationship. Science. 2023;379(6633):eabp8964. doi: 10.1126/science.abp8964 36795835

[24] Panocha D, Roet JEG, Kuipers JE, et al. Lymph node fibroblast-produced extracellular matrix shapes immune function. Trends Immunol. 2025;46(3):229–243. doi: 10.1016/j.it.2025.02.002 40023738

[25] Basu R, Huse M. Mechanical communication at the immunological synapse. Trends Cell Biol. 2017;27(4):241–254. doi: 10.1016/j.tcb.2016.10.005 27986534 PMC5367987

[26] Huse M. Mechanoregulation of lymphocyte cytotoxicity. Nat Rev Immunol. 2025;25(9):680–695. doi: 10.1038/s41577-025-01173-2 40312550

[27] Huse M. Mechanical forces in the immune system. Nat Rev Immunol. 2017;17(11):679–690. doi: 10.1038/nri.2017.74 28757604 PMC6312705

[28] Zhu C, Chen W, Lou J, et al. Mechanosensing through immunoreceptors. Nat Immunol. 2019;20(10):1269–1278. doi: 10.1038/s41590-019-0491-1 31534240 PMC7592628

[29] Bieling P, Li T-D, Weichsel J, et al. Force feedback controls motor activity and mechanical properties of self-assembling branched actin networks. Cell. 2016;164(1–2):115–127. doi: 10.1016/j.cell.2015.11.057 26771487 PMC5033619

[30] Starr DA, Fridolfsson HN. Interactions between nuclei and the cytoskeleton are mediated by SUN-KASH nuclear-envelope bridges. Annu Rev Cell Dev Biol. 2010;26(1):421–444. doi: 10.1146/annurev-cellbio-100109-104037 20507227 PMC4053175

[31] Luxton GWG, Gomes ER, Folker ES, et al. Linear arrays of nuclear envelope proteins harness retrograde actin flow for nuclear movement. Science. 2010;329(5994):956–959. doi: 10.1126/science.1189072 20724637 PMC3938394

[32] Luxton GWG, Gomes ER, Folker ES, et al. Tan lines. Nucleus. 2011;2(3):173–181. doi: 10.4161/nucl.2.3.16243 21818410 PMC3149877

[33] Moreau HD, Piel M, Voituriez R, et al. Integrating physical and molecular insights on immune cell migration. Trends Immunol. 2018;39(8):632–643. doi: 10.1016/j.it.2018.04.007 29779848

[34] Lämmermann T, Sixt M. Mechanical modes of “amoeboid” cell migration. Curr Opin Cell Biol. 2009;21(5):636–644. doi: 10.1016/j.ceb.2009.05.003 19523798

[35] Yamada KM, Sixt M. Mechanisms of 3d cell migration. Nat Rev Mol Cell Biol. 2019;20(12):738–752. doi: 10.1038/s41580-019-0172-9 31582855

[36] Dong C, Skalak R, Sung KL. Cytoplasmic rheology of passive neutrophils. BIR. 1991;28(6):557–567. doi: 10.3233/BIR-1991-28607 1818744

[37] Wolf K, Te Lindert M, Krause M, et al. Physical limits of cell migration: control by ECM space and nuclear deformation and tuning by proteolysis and traction force. J Cell Biol. 2013;201(7):1069–1084. doi: 10.1083/jcb.201210152 23798731 PMC3691458

[38] Harada T, Swift J, Irianto J, et al. Nuclear lamin stiffness is a barrier to 3D migration, but softness can limit survival. J Cell Biol. 2014;204(5):669–682. doi: 10.1083/jcb.201308029 24567359 PMC3941057

[39] Ramoser H, Laurain V, Bischof H, et al. Leukocyte segmentation and classification in blood-smear images. Conf Proc IEEE Eng Med Biol Soc. 2005;2005:3371–3374. doi: 10.1109/IEMBS.2005.1617200 17280945

[40] Prinyakupt J, Pluempitiwiriyawej C. Segmentation of white blood cells and comparison of cell morphology by linear and naïve Bayes classifiers. Biomed Eng Online. 2015;14(1):63. doi: 10.1186/s12938-015-0037-1 26123131 PMC4485641

[41] Mosser DM, Edwards JP. Exploring the full spectrum of macrophage activation. Nat Rev Immunol. 2008;8(12):958–969. doi: 10.1038/nri2448 19029990 PMC2724991

[42] Skinner BM, Johnson EEP. Nuclear morphologies: their diversity and functional relevance. Chromosoma. 2017;126(2):195–212. doi: 10.1007/s00412-016-0614-5 27631793 PMC5371643

[43] Whitmore LC, Weems MN, Allen L-A. Cutting edge: helicobacter pylori induces nuclear hypersegmentation and subtype differentiation of human neutrophils in vitro. J Immunol. 2017;198(5):1793–1797. doi: 10.4049/jimmunol.1601292 28148734 PMC5318842

[44] Manley HR, Keightley MC, Lieschke GJ. The neutrophil nucleus: an important influence on neutrophil migration and function. Front Immunol. 2018;9:2867. doi: 10.3389/fimmu.2018.02867 30564248 PMC6288403

[45] Silva-Del Toro SL, Allen L-A. Microtubules and dynein regulate human neutrophil nuclear volume and hypersegmentation during H. pylori infection. Front Immunol. 2021;12:653100. doi: 10.3389/fimmu.2021.653100 33828562 PMC8019731

[46] Olins AL, Olins DE. Cytoskeletal influences on nuclear shape in granulocytic HL-60 cells. BMC Cell Biol. 2004;5(1):30. doi: 10.1186/1471-2121-5-30 15317658 PMC516025

[47] Liu J, Li Z, Li M, et al. Vimentin regulates nuclear segmentation in neutrophils. Proc Natl Acad Sci U S A. 2023;120(48):e2307389120. doi: 10.1073/pnas.2307389120 37983515 PMC10691343

[48] Ulloa R, Corrales O, Cabrera-Reyes F, et al. B cells adapt their nuclear morphology to organize the immune synapse and facilitate antigen extraction. Front Immunol. 2022;12:801164. doi: 10.3389/fimmu.2021.801164 PMC8863768 35222354

[49] Kroll J, Hauschild R, Kuznetcov A, et al. Adaptive pathfinding by nucleokinesis during amoeboid migration. EMBO J. 2023;42(24):e114557. doi: 10.15252/embj.2023114557 37987147 PMC10711653

[50] Shen C, Mulder E, Buitenwerf W, et al. Nuclear segmentation facilitates neutrophil migration. J Cell Sci. 2023;136(11):jcs260768. doi: 10.1242/jcs.260768 37288767 PMC10309577

[51] Rowat AC, Jaalouk DE, Zwerger M, et al. Nuclear envelope composition determines the ability of neutrophil-type cells to passage through micron-scale constrictions. J Biol Chem. 2013;288(12):8610–8618. doi: 10.1074/jbc.M112.441535 23355469 PMC3605679

[52] Wolf K, Alexander S, Schacht V, et al. Collagen-based cell migration models in vitro and in vivo. Semin Cell Dev Biol. 2009;20(8):931–941. doi: 10.1016/j.semcdb.2009.08.005 19682592 PMC4021709

[53] Venturini V, Pezzano F, Català Castro F, et al. The nucleus measures shape changes for cellular proprioception to control dynamic cell behavior. Science. 2020;370(6514):eaba2644. doi: 10.1126/science.aba2644 33060331

[54] Lin W, Wang S, Liu R, et al. Research progress of cPLA2 in cardiovascular diseases (Review). Mol Med Rep. 2025;31(4):103. doi: 10.3892/mmr.2025.13468 39981923 PMC11868774

[55] Liu Y-J, Le Berre M, Lautenschlaeger F, et al. Confinement and low adhesion induce fast amoeboid migration of slow mesenchymal cells. Cell. 2015;160(4):659–672. doi: 10.1016/j.cell.2015.01.007 25679760

[56] Ruprecht V, Wieser S, Callan-Jones A, et al. Cortical contractility triggers a stochastic switch to fast amoeboid cell motility. Cell. 2015;160(4):673–685. doi: 10.1016/j.cell.2015.01.008 25679761 PMC4328143

[57] Guilak F, Tedrow JR, Burgkart R. Viscoelastic properties of the cell nucleus. Biochem Biophys Res Commun. 2000;269(3):781–786. doi: 10.1006/bbrc.2000.2360 10720492

[58] Karling T, Weavers H. Immune cells adapt to confined environments in vivo to optimise nuclear plasticity for migration. EMBO Rep. 2025;26(5):1238–1268. doi: 10.1038/s44319-025-00381-0 39915297 PMC11894099

[59] Shin J-W, Spinler KR, Swift J, et al. Lamins regulate cell trafficking and lineage maturation of adult human hematopoietic cells. Proc Natl Acad Sci U S A. 2013;110(47):18892–18897. doi: 10.1073/pnas.1304996110 24191023 PMC3839750

[60] Li Y, Chen M, Chang W. Roles of the nucleus in leukocyte migration. J Leukoc Biol. 2022;112(4):771–783. doi: 10.1002/JLB.1MR0622-473RR 35916042

[61] Salvermoser M, Begandt D, Alon R, et al. Nuclear deformation during neutrophil migration at sites of inflammation. Front Immunol. 2018;9:2680. doi: 10.3389/fimmu.2018.02680 30505310 PMC6250837

[62] Estabrook ID, Thiam HR, Piel M, et al. Calculation of the force field required for nucleus deformation during cell migration through constrictions. PLOS Comput Biol. 2021;17(5):e1008592. doi: 10.1371/journal.pcbi.1008592 34029312 PMC8177636

[63] Thompson SB, Sandor AM, Lui V, et al. Formin-like 1 mediates effector T cell trafficking to inflammatory sites to enable T cell-mediated autoimmunity. Elife. 2020;9:e58046. doi: 10.7554/eLife.58046 32510333 PMC7308091

[64] Lämmermann T, Bader BL, Monkley SJ, et al. Rapid leukocyte migration by integrin-independent flowing and squeezing. Nature. 2008;453(7191):51–55. doi: 10.1038/nature06887 18451854

[65] Barbier L, Sáez PJ, Attia R, et al. Myosin II activity is selectively needed for migration in highly confined microenvironments in mature dendritic cells. Front Immunol. 2019;10:747. doi: 10.3389/fimmu.2019.00747 31031752 PMC6474329

[66] Jacobelli J, Estin Matthews M, Chen S, et al. Activated T cell trans-endothelial migration relies on myosin-IIA contractility for squeezing the cell nucleus through endothelial cell barriers. PLOS ONE. 2013;8(9):e75151. doi: 10.1371/journal.pone.0075151 24069389 PMC3777879

[67] Krummel MF, Friedman RS, Jacobelli J. Modes and mechanisms of T cell motility: roles for confinement and myosin-IIA. Curr Opin Cell Biol. 2014;30:9–16. doi: 10.1016/j.ceb.2014.05.003 24905977 PMC4178009

[68] Salvermoser M, Pick R, Weckbach LT, et al. Myosin 1f is specifically required for neutrophil migration in 3D environments during acute inflammation. Blood. 2018;131(17):1887–1898. doi: 10.1182/blood-2017-10-811851 29487067

[69] Su HC. Dedicator of cytokinesis 8 (DOCK8) deficiency. Curr Opin Allergy Clin Immunol. 2010;10(6):515–520. doi: 10.1097/ACI.0b013e32833fd718 20864884 PMC3096565

[70] Shen C, Cerf A, Postat J, et al. A Dock8-dependent mechanosensitive central actin pool maintains T cell shape and protects the nucleus during migration. Sci Immunol. 2025;10(109):eadt9239. doi: 10.1126/sciimmunol.adt9239 40570086

[71] Reis-Rodrigues P, Avellaneda MJ, Canigova N, et al. Migrating immune cells globally coordinate protrusive forces. Nat Immunol. 2025;26(8):1258–1266. doi: 10.1038/s41590-025-02211-w 40664976 PMC12307229

[72] Pflicke H, Sixt M. Preformed portals facilitate dendritic cell entry into afferent lymphatic vessels. J Exp Med. 2009;206(13):2925–2935. doi: 10.1084/jem.20091739 19995949 PMC2806476

[73] Gaertner F, Reis-Rodrigues P, de Vries I, et al. WASp triggers mechanosensitive actin patches to facilitate immune cell migration in dense tissues. Dev Cell. 2022;57(1):47–62.e9. doi: 10.1016/j.devcel.2021.11.024 34919802 PMC8751638

[74] Barzilai S, Yadav SK, Morrell S, et al. Leukocytes breach endothelial barriers by insertion of nuclear lobes and disassembly of endothelial actin filaments. Cell Rep. 2017;18(3):685–699. doi: 10.1016/j.celrep.2016.12.076 28099847

[75] Kumar S, Das A, Sen S. Multicompartment cell-based modeling of confined migration: regulation by cell intrinsic and extrinsic factors. MBoC. 2018;29(13):1599–1610. doi: 10.1091/mbc.E17-05-0313 29718766 PMC6080655

[76] Raab M, Gentili M, de Belly H, et al. Escrt III repairs nuclear envelope ruptures during cell migration to limit DNA damage and cell death. Science. 2016;352(6283):359–362. doi: 10.1126/science.aad7611 27013426

[77] Denais CM, Gilbert RM, Isermann P, et al. Nuclear envelope rupture and repair during cancer cell migration. Science. 2016;352(6283):353–358. doi: 10.1126/science.aad7297 27013428 PMC4833568

[78] Schmitt MT, Kroll J, Ruiz-Fernandez MJA, et al. Protecting centrosomes from fracturing enables efficient cell navigation. Sci Adv. 2025;11(17):eadx4047. doi: 10.1126/sciadv.adx4047 40279414 PMC12024656

[79] Nourshargh S, Hordijk PL, Sixt M. Breaching multiple barriers: leukocyte motility through venular walls and the interstitium. Nat Rev Mol Cell Biol. 2010;11(5):366–378. doi: 10.1038/nrm2889 20414258

[80] Hadjitheodorou A, Bell GRR, Ellett F, et al. Directional reorientation of migrating neutrophils is limited by suppression of receptor input signaling at the cell rear through myosin II activity. Nat Commun. 2021;12(1):6619. doi: 10.1038/s41467-021-26622-z 34785640 PMC8595366

[81] Hadjitheodorou A, Bell GRR, Ellett F, et al. Leading edge competition promotes context-dependent responses to receptor inputs to resolve directional dilemmas in neutrophil migration. Cell Syst. 2023;14(3):196–209.e6. doi: 10.1016/j.cels.2023.02.001 36827986 PMC10150694

[82] Hons M, Kopf A, Hauschild R, et al. Chemokines and integrins independently tune actin flow and substrate friction during intranodal migration of T cells. Nat Immunol. 2018;19(6):606–616. doi: 10.1038/s41590-018-0109-z 29777221

[83] Leithner A, Eichner A, Müller J, et al. Diversified actin protrusions promote environmental exploration but are dispensable for locomotion of leukocytes. Nat Cell Biol. 2016;18(11):1253–1259. doi: 10.1038/ncb3426 27775702

[84] Zhang Q, Dove CG, Hor JL, et al. Dock8 regulates lymphocyte shape integrity for skin antiviral immunity. J Exp Med. 2014;211(13):2549–2566. doi: 10.1084/jem.20141307 25422492 PMC4267229

[85] Harada Y, Tanaka Y, Terasawa M, et al. Dock8 is a Cdc42 activator critical for interstitial dendritic cell migration during immune responses. Blood. 2012;119(19):4451–4461. doi: 10.1182/blood-2012-01-407098 22461490 PMC3418773

[86] Sarris M, Sixt M. Navigating in tissue mazes: chemoattractant interpretation in complex environments. Curr Opin Cell Biol. 2015;36:93–102. doi: 10.1016/j.ceb.2015.08.001 26355911

[87] Kroll J, Renkawitz J. Principles of organelle positioning in motile and non-motile cells. EMBO Rep. 2024;25(5):2172–2187. doi: 10.1038/s44319-024-00135-4 38627564 PMC11094012

[88] Hale BD, Severin Y, Graebnitz F, et al. Cellular architecture shapes the naïve T cell response. Science. 2024;384(6700):eadh8697. doi: 10.1126/science.adh8967 38843327

[89] Lammerding J, Schulze PC, Takahashi T, et al. Lamin A/C deficiency causes defective nuclear mechanics and mechanotransduction. J Clin Invest. 2004;113(3):370–378. doi: 10.1172/JCI200419670 14755334 PMC324542

[90] Sullivan T, Escalante-Alcalde D, Bhatt H, et al. Loss of a-type lamin expression compromises nuclear envelope integrity leading to muscular dystrophy. J Cell Biol. 1999;147(5):913–920. doi: 10.1083/jcb.147.5.913 10579712 PMC2169344

[91] Hale JS, Frock RL, Mamman SA, et al. Cell-extrinsic defective lymphocyte development in Lmna(-/-) mice. PLOS ONE. 2010;5(4):e10127. doi: 10.1371/journal.pone.0010127 20405040 PMC2853576

[92] González-Granado JM, Silvestre-Roig C, Rocha-Perugini V, et al. Nuclear envelope lamin-A couples actin dynamics with immunological synapse architecture and T cell activation. Sci Signal. 2014;7(322):ra37–ra37. doi: 10.1126/scisignal.2004872 24757177 PMC4337980

[93] Toribio-Fernández R, Zorita V, Rocha-Perugini V, et al. Lamin A/C augments Th1 differentiation and response against vaccinia virus and Leishmania major. Cell Death Dis. 2018;9(1):9. doi: 10.1038/s41419-017-0007-6 29311549 PMC5849043

[94] Kaech SM, Wherry EJ, Ahmed R. Effector and memory T-cell differentiation: implications for vaccine development. Nat Rev Immunol. 2002;2(4):251–262. doi: 10.1038/nri778 12001996

[95] Mehl JL, Earle A, Lammerding J, et al. Blockage of lamin-A/C loss diminishes the pro-inflammatory macrophage response. iScience. 2022;25(12):105528. doi: 10.1016/j.isci.2022.105528 36465100 PMC9708799

[96] Jiao S, Li C, Guo F, et al. Sun1/2 controls macrophage polarization via modulating nuclear size and stiffness. Nat Commun. 2023;14(1):6416. doi: 10.1038/s41467-023-42187-5 37828059 PMC10570371

[97] Fu Y, Jing Z, Chen T, et al. Nanotube patterning reduces macrophage inflammatory response via nuclear mechanotransduction. J Nanobiotechnol. 2023;21(1):229. doi: 10.1186/s12951-023-01912-4 PMC10354937 37468894

[98] Kim Y, Bayona PW, Kim M, et al. Macrophage lamin A/C regulates inflammation and the development of obesity-induced insulin resistance. Front Immunol. 2018;9:696. doi: 10.3389/fimmu.2018.00696 29731750 PMC5920030

[99] Herrero-Fernández B, Ortega-Zapero M, Gómez-Bris R, et al. Role of lamin A/C on dendritic cell function in antiviral immunity. Cell Mol Life Sci. 2024;81(1):400. doi: 10.1007/s00018-024-05423-9 39264480 PMC11393282

[100] Wang B, Bian Q. Regulation of 3D genome organization during T cell activation. FEBS J. 2025;292(8):1833–1852. doi: 10.1111/febs.17211 38944686

[101] Rawlings JS, Gatzka M, Thomas PG, et al. Chromatin condensation via the condensin II complex is required for peripheral T-cell quiescence. EMBO J. 2011;30(2):263–276. doi: 10.1038/emboj.2010.314 21169989 PMC3025460

[102] Gate RE, Cheng CS, Aiden AP, et al. Genetic determinants of co-accessible chromatin regions in activated T cells across humans. Nat Genet. 2018;50(8):1140–1150. doi: 10.1038/s41588-018-0156-2 29988122 PMC6097927

[103] Allan RS, Zueva E, Cammas F, et al. An epigenetic silencing pathway controlling T helper 2 cell lineage commitment. Nature. 2012;487(7406):249–253. doi: 10.1038/nature11173 22763435

[104] Isoda T, Moore AJ, He Z, et al. Non-coding transcription instructs chromatin folding and compartmentalization to dictate enhancer-promoter communication and T cell fate. Cell. 2017;171(1):103–119.e18. doi: 10.1016/j.cell.2017.09.001 28938112 PMC5621651

[105] Barutcu AR, Lajoie BR, McCord RP, et al. Chromatin interaction analysis reveals changes in small chromosome and telomere clustering between epithelial and breast cancer cells. Genome Biol. 2015;16(1):214. doi: 10.1186/s13059-015-0768-0 26415882 PMC4587679

[106] Zhang Y, An L, Xu J, et al. Enhancing Hi-C data resolution with deep convolutional neural network HiCPlus. Nat Commun. 2018;9(1):750. doi: 10.1038/s41467-018-03113-2 29467363 PMC5821732

[107] Suarez IAR, Pathni A, Fazekas F, et al. Nuclear morphology and chromatin organization modulate T cell cytoskeletal remodeling and immune synapse formation. BioRxiv. 2025;2025.08.25.672234. doi: 10.1101/2025.08.25.672234 PMC13525613 42418713

[108] Stein GS, Montecino M, van Wijnen AJ, et al. Nuclear structure-gene expression interrelationships: implications for aberrant gene expression in cancer. Cancer Res. 2000;60(8):2067–2076. PMID: 10786661. 10786661

[109] Takei H, Araki A, Watanabe H, et al. Rapid killing of human neutrophils by the potent activator phorbol 12-myristate 13-acetate (PMA) accompanied by changes different from typical apoptosis or necrosis. J Leukoc Biol. 1996;59:229–240. doi: 10.1002/jlb.59.2.229 8603995

[110] Brinkmann V, Reichard U, Goosmann C, et al. Neutrophil extracellular traps kill bacteria. Science. 2004;303(5663):1532–1535. doi: 10.1126/science.1092385 15001782

[111] Huang S-S, O’Sullivan KM. The expanding role of extracellular traps in inflammation and autoimmunity: the new players in casting dark webs. IJMS. 2022;23(7):3793. doi: 10.3390/ijms23073793 35409152 PMC8998317

[112] Thanabalasuriar A, Scott BNV, Peiseler M, et al. Neutrophil extracellular traps confine Pseudomonas aeruginosa ocular biofilms and restrict brain invasion. Cell Host Microbe. 2019;25(4):526–536.e4. doi: 10.1016/j.chom.2019.02.007 30930127 PMC7364305

[113] Varjú I, Kolev K. Networks that stop the flow: a fresh look at fibrin and neutrophil extracellular traps. Thromb Res. 2019;182:1–11. doi: 10.1016/j.thromres.2019.08.003 31415922

[114] Zhou Y, Xu Z, Liu Z. Impact of neutrophil extracellular traps on thrombosis formation: new findings and future perspective. Front Cell Infect Microbiol. 2022;12:910908. doi: 10.3389/fcimb.2022.910908 35711663 PMC9195303

[115] Jaboury S, Wang K, O’Sullivan KM, et al. Netosis as an oncologic therapeutic target: a mini review. Front Immunol. 2023;14:1170603. doi: 10.3389/fimmu.2023.1170603 37143649 PMC10151565

[116] Thiam HR, Wong SL, Qiu R, et al. Netosis proceeds by cytoskeleton and endomembrane disassembly and PAD4-mediated chromatin decondensation and nuclear envelope rupture. Proc Natl Acad Sci USA. 2020;117(13):7326–7337. doi: 10.1073/pnas.1909546117 PMC7132277 32170015

[117] Fuchs TA, Abed U, Goosmann C, et al. Novel cell death program leads to neutrophil extracellular traps. J Cell Biol. 2007;176:231–241. doi: 10.1083/jcb.200606027 17210947 PMC2063942

[118] Clark SR, Ma AC, Tavener SA, et al. Platelet TLR4 activates neutrophil extracellular traps to ensnare bacteria in septic blood. Nat Med. 2007;13:463–469. doi: 10.1038/nm1565 17384648

[119] Yipp BG, Petri B, Salina D, et al. Infection-induced NETosis is a dynamic process involving neutrophil multitasking in vivo. Nat Med. 2012;18:1386–1393. doi: 10.1038/nm.2847 22922410 PMC4529131

[120] Arya SB, Collie SP, Xu Y, et al. Neutrophils secrete exosome-associated DNA to resolve sterile acute inflammation. Nat Cell Biol. 2025;27:931–947. doi: 10.1038/s41556-025-01671-4 40404894 PMC12206380

[121] Khan MA, Palaniyar N. Transcriptional firing helps to drive netosis. Sci Rep. 2017;7(1):41749. doi: 10.1038/srep41749 28176807 PMC5296899

[122] Kenny EF, Herzig A, Krüger R, et al. Diverse stimuli engage different neutrophil extracellular trap pathways. Elife. 2017;6:e24437. doi: 10.7554/eLife.24437 28574339 PMC5496738

[123] Chen X, Shen Y, Draper W, et al. Atac-see reveals the accessible genome by transposase-mediated imaging and sequencing. Nat Methods. 2016;13(12):1013–1020. doi: 10.1038/nmeth.4031 27749837 PMC5509561

[124] Neubert E, Meyer D, Rocca F, et al. Chromatin swelling drives neutrophil extracellular trap release. Nat Commun. 2018;9(1):3767. doi: 10.1038/s41467-018-06263-5 30218080 PMC6138659

[125] Wang Y, Li M, Stadler S, et al. Histone hypercitrullination mediates chromatin decondensation and neutrophil extracellular trap formation. J Cell Biol. 2009;184(2):205–213. doi: 10.1083/jcb.200806072 19153223 PMC2654299

[126] Papayannopoulos V, Metzler KD, Hakkim A, et al. Neutrophil elastase and myeloperoxidase regulate the formation of neutrophil extracellular traps. J Cell Biol. 2010;191:677–691. doi: 10.1083/jcb.201006052 20974816 PMC3003309

[127] Hamam HJ, Khan MA, Palaniyar N. Histone acetylation promotes neutrophil extracellular trap formation. Biomolecules. 2019;9:32. doi: 10.3390/biom9010032 30669408 PMC6359456

[128] Teijeira Á, Garasa S, Gato M, et al. Cxcr1 and cxcr2 chemokine receptor agonists produced by tumors induce neutrophil extracellular traps that interfere with immune cytotoxicity. Immunity. 2020;52:856–871.e8. doi: 10.1016/j.immuni.2020.03.001 32289253

[129] Enyedi B, Niethammer P. Nuclear membrane stretch and its role in mechanotransduction. Nucleus. 2017;8:156–161. doi: 10.1080/19491034.2016.1263411 28112995 PMC5403133

[130] Simon C, Caorsi V, Campillo C, et al. Interplay between membrane tension and the actin cytoskeleton determines shape changes. Phys Biol. 2018;15:065004. doi: 10.1088/1478-3975/aad1ab 29978835

[131] Raucher D, Sheetz MP. Characteristics of a membrane reservoir buffering membrane tension. Biophys J. 1999;77:1992–2002. doi: 10.1016/S0006-3495(99)77040-2 10512819 PMC1300480

[132] Stewart MP, Helenius J, Toyoda Y, et al. Hydrostatic pressure and the actomyosin cortex drive mitotic cell rounding. Nature. 2011;469(7329):226–230. doi: 10.1038/nature09642 21196934

[133] Shen Z, Gelashvili Z, Niethammer P. Buffering of nuclear membrane tension and mechanotransduction by the endoplasmic reticulum revealed by quantitative ALPIN imaging. Res Sq. 2024:rs.3.rs–5530637. doi: 10.21203/rs.3.rs-5530637/v1

[134] Amulic B, Knackstedt SL, Abu Abed U, et al. Cell-cycle proteins control production of neutrophil extracellular traps. Dev Cell. 2017;43(4):449–462.e5. doi: 10.1016/j.devcel.2017.10.013 29103955

[135] Li Y, Li M, Weigel B, et al. Nuclear envelope rupture and NET formation is driven by PKCα-mediated lamin B disassembly. EMBO Rep. 2020;21(8):e48779. doi: 10.15252/embr.201948779 32537912 PMC7403722

[136] Singh J, Zlatar L, Muñoz-Becerra M, et al. Calpain-1 weakens the nuclear envelope and promotes the release of neutrophil extracellular traps. Cell Commun Signaling. 2024;22:435. doi: 10.1186/s12964-024-01785-6 PMC11384698 39252008

[137] Pilsczek FH, Salina D, Poon KKH, et al. A novel mechanism of rapid nuclear neutrophil extracellular trap formation in response to Staphylococcus aureus. J Immunol. 2010;185(12):7413–7425. doi: 10.4049/jimmunol.1000675 21098229

[138] Morival J, Hazelwood A, Lammerding J. Feeling the force from within - new tools and insights into nuclear mechanotransduction. J Cell Sci. 2025;138(5):JCS263615. doi: 10.1242/jcs.263615 40059756 PMC11959624

[139] Lombardi ML, Lammerding J. Keeping the LINC: the importance of nucleocytoskeletal coupling in intracellular force transmission and cellular function. Biochem Soc Trans. 2011;39(6):1729–1734. doi: 10.1042/BST20110686 22103516 PMC4589539

[140] Giverso C, Grillo A, Preziosi L. Influence of nucleus deformability on cell entry into cylindrical structures. Biomech Model Mechanobiol. 2014;13(3):481–502. doi: 10.1007/s10237-013-0510-3 23838726

[141] Pajerowski JD, Dahl KN, Zhong FL, et al. Physical plasticity of the nucleus in stem cell differentiation. Proc Natl Acad Sci USA; 2007;104(40):15619–15624. doi: 10.1073/pnas.0702576104 PMC2000408 17893336

[142] Dahl KN, Kahn SM, Wilson KL, et al. The nuclear envelope lamina network has elasticity and a compressibility limit suggestive of a molecular shock absorber. J Cell Sci. 2004;117(20):4779–4786. doi: 10.1242/jcs.01357 15331638

[143] Swift J, Ivanovska IL, Buxboim A, et al. Nuclear lamin-A scales with tissue stiffness and enhances matrix-directed differentiation. Science. 2013;341(6149):1240104. doi: 10.1126/science.1240104 23990565 PMC3976548

[144] Buxboim A, Swift J, Irianto J, et al. Matrix elasticity regulates lamin-A,C phosphorylation and turnover with feedback to actomyosin. Curr Biol. 2014;24(16):1909–1917. doi: 10.1016/j.cub.2014.07.001 25127216 PMC4373646

[145] Stephens AD, Banigan EJ, Adam SA, et al. Chromatin and lamin A determine two different mechanical response regimes of the cell nucleus. MBoC. 2017;28(14):1984–1996. doi: 10.1091/mbc.e16-09-0653 28057760 PMC5541848

[146] Grevesse T, Dabiri BE, Parker KK, et al. Opposite rheological properties of neuronal microcompartments predict axonal vulnerability in brain injury. Sci Rep. 2015;5(1):9475. doi: 10.1038/srep09475 25820512 PMC4377573

[147] Guilluy C, Osborne LD, Van Landeghem L, et al. Isolated nuclei adapt to force and reveal a mechanotransduction pathway in the nucleus. Nat Cell Biol. 2014;16(4):376–381. doi: 10.1038/ncb2927 24609268 PMC4085695

[148] Röber RA, Sauter H, Weber K, et al. Cells of the cellular immune and hemopoietic system of the mouse lack lamins A/C: distinction versus other somatic cells. J Cell Sci. 1990;95(4):587–598. doi: 10.1242/jcs.95.4.587 2200797

[149] Paulin-Levasseur M, Scherbarth A, Traub U, et al. Lack of lamins A and C in mammalian hemopoietic cell lines devoid of intermediate filament proteins. Eur J Cell Biol. 1988;47(1):121–131. PMID: 3068054. 3068054

[150] Guilly MN, Bensussan A, Bourge JF, et al. A human T lymphoblastic cell line lacks lamins A and C. EMBO J. 1987;6(12):3795–3799. doi: 10.1002/j.1460-2075.1987.tb02715.x 3501373 PMC553851

[151] Stadelmann B, Khandjian E, Hirt A, et al. Repression of nuclear lamin A and C gene expression in human acute lymphoblastic leukemia and non-Hodgkin’s lymphoma cells. Leuk Res. 1990;14(9):815–821. doi: 10.1016/0145-2126(90)90076-L 2232854

[152] Guilly MN, Kolb JP, Gosti F, et al. Lamins A and C are not expressed at early stages of human lymphocyte differentiation. Exp Cell Res. 1990;189(1):145–147. doi: 10.1016/0014-4827(90)90267-E 2347374

[153] Guzniczak E, Mohammad Zadeh M, Dempsey F, et al. High-throughput assessment of mechanical properties of stem cell derived red blood cells, toward cellular downstream processing. Sci Rep. 2017;7(1):14457. doi: 10.1038/s41598-017-14958-w 29089557 PMC5663858

[154] Olins AL, Herrmann H, Lichter P, et al. Nuclear envelope and chromatin compositional differences comparing undifferentiated and retinoic acid- and phorbol ester-treated HL-60 cells. Exp Cell Res. 2001;268(2):115–127. doi: 10.1006/excr.2001.5269 11478838

[155] Olins AL, Zwerger M, Herrmann H, et al. The human granulocyte nucleus: unusual nuclear envelope and heterochromatin composition. Eur J Cell Biol. 2008;87(5):279–290. doi: 10.1016/j.ejcb.2008.02.007 18396345 PMC2438038

[156] Jung-Garcia Y, Maiques O, Monger J, et al. Lap1 supports nuclear adaptability during constrained melanoma cell migration and invasion. Nat Cell Biol. 2023;25(1):108–119. doi: 10.1038/s41556-022-01042-3 36624187 PMC9859759

[157] Chen NY, Kim PH, Tu Y, et al. Increased expression of LAP2β eliminates nuclear membrane ruptures in nuclear lamin–deficient neurons and fibroblasts. Proc Natl Acad Sci USA 2021;118(25):e2107770118. doi: 10.1073/pnas.2107770118 PMC8237679 34161290

[158] Olins AL, Hoang TV, Zwerger M, et al. The LINC-less granulocyte nucleus. Eur J Cell Biol. 2009;88(4):203–214. doi: 10.1016/j.ejcb.2008.10.001 19019491 PMC2671807

[159] Lityagina O, Dobreva G. The LINC between mechanical forces and chromatin. Front Physiol. 2021;12:710809. doi: 10.3389/fphys.2021.710809 34408666 PMC8365421

[160] Lombardi ML, Lammerding J. Keeping the LINC: the importance of nucleo-cytoskeletal coupling in intracellular force transmission and cellular function. Biochem Soc Trans. 2011;39:1729–1734. doi: 10.1042/BST20110686 22103516 PMC4589539

[161] Petrovic S, Mobbs GW, Hoelz A. Structure, function and assembly of nuclear pore complexes. Nat Rev Mol Cell Biol. 2025;1–20. doi: 10.1038/s41580-025-00881-w 40926106 PMC13242641

[162] Singer SJ, Nicolson GL. The fluid mosaic model of the structure of cell membranes. Science. 1972;175:720–731. doi: 10.1126/science.175.4023.720 4333397

[163] Nicolson GL, Ferreira de Mattos G. The fluid-mosaic model of cell membranes: a brief introduction, historical features, some general principles, and its adaptation to current information. Biochim Biophys Acta Biomembr. 2023;1865:184135. doi: 10.1016/j.bbamem.2023.184135 36746313

[164] Keren K. Cell motility: the integrating role of the plasma membrane. Eur Biophys J. 2011;40:1013–1027. doi: 10.1007/s00249-011-0741-0 21833780 PMC3158336

[165] Enyedi B, Jelcic M, Niethammer P. The cell nucleus serves as a mechanotransducer of tissue damage-induced inflammation. Cell. 2016;165:1160–1170. doi: 10.1016/j.cell.2016.04.016 27203112 PMC4875569

[166] Agrawal A, Lele TP. Geometry of the nuclear envelope determines its flexural stiffness. Mol Biol Cell. 2020;31:1815–1821. doi: 10.1091/mbc.E20-02-0163 32583742 PMC7521844

[167] Tsai P-L, Zhao C, Turner E, et al. The lamin B receptor is essential for cholesterol synthesis and perturbed by disease-causing mutations. Elife. 2016;5:e16011. doi: 10.7554/eLife.16011 27336722 PMC4951196

[168] Turner EM, Schlieker C. Pelger-Huët anomaly and Greenberg skeletal dysplasia: lBR-associated diseases of cholesterol metabolism. Rare Dis. 2016;4:e1241363. doi: 10.1080/21675511.2016.1241363 27830109 PMC5077067

[169] Jiang J, Tu H, Li P. Lipid metabolism and neutrophil function. Cell Immunol. 2022;377:104546. doi: 10.1016/j.cellimm.2022.104546 35688009

[170] Uchańska A, Morytko A, Kwiecień K, et al. Lazy neutrophils - a lack of DGAT1 reduces the chemotactic activity of mouse neutrophils. Inflamm Res. 2024;73:1631–1643. doi: 10.1007/s00011-024-01920-6 39043892 PMC11445369

[171] Uhlén M, Fagerberg L, Hallström BM, et al. Tissue-based map of the human proteome. Science. 2015;347:1260419. doi: 10.1126/science.1260419 25613900

[172] Yen C-L, Stone SJ, Koliwad S, et al. Dgat enzymes and triacylglycerol biosynthesis. J Lipid Res. 2008;49:2283–2301. doi: 10.1194/jlr.R800018-JLR200 18757836 PMC3837458

[173] Nørregaard R, Kwon T-H, Frøkiær J. Physiology and pathophysiology of cyclooxygenase-2 and prostaglandin E2 in the kidney. Kidney Res Clin Pract. 2015;34:194–200. doi: 10.1016/j.krcp.2015.10.004 26779421 PMC4688592

[174] Lutkewitte AJ, Finck BN. Regulation of signaling and metabolism by lipin-mediated phosphatidic acid phosphohydrolase activity. Biomolecules. 2020;10:1386. doi: 10.3390/biom10101386 33003344 PMC7600782

[175] Baltoumas FA, Sofras D, Apostolakou AE, et al. NucEnvDB: a database of nuclear envelope proteins and their interactions. Membranes (Basel). 2023;13:62. doi: 10.3390/membranes13010062 36676869 PMC9861991

[176] Kato N, Ishijima A, Inaba T, et al. Effects of lipid composition and solution conditions on the mechanical properties of membrane vesicles. Membranes (Basel). 2015;5:22–47. doi: 10.3390/membranes5010022 25611306 PMC4384090

[177] Cockcroft S. Mammalian lipids: structure, synthesis and function. Essays Biochem. 2021;65:813–845. doi: 10.1042/EBC20200067 34415021 PMC8578989

[178] Iglesias-Artola JM, Böhlig K, Schuhmann K, et al. Quantitative imaging of lipid transport in mammalian cells. Nature. 2025 Oct;646(8084):474–482. doi: 10.1039/b920629a PMC12507682 40836094

[179] Gracià RS, Bezlyepkina N, Knorr RL, et al. Effect of cholesterol on the rigidity of saturated and unsaturated membranes: fluctuation and electrodeformation analysis of giant vesicles. Soft Matter. 2010;6:1472–1482. doi: 10.1039/b920629a

[180] Pöhnl M, Trollmann MFW, Böckmann RA. Nonuniversal impact of cholesterol on membranes mobility, curvature sensing and elasticity. Nat Commun. 2023;14:8038. doi: 10.1038/s41467-023-43892-x 38081812 PMC10713574

[181] Pan J, Mills TT, Tristram-Nagle S, et al. Cholesterol perturbs lipid bilayers nonuniversally. Phys Rev Lett. 2008;100:198103. doi: 10.1103/PhysRevLett.100.198103 18518492 PMC2695669

[182] Henriksen J, Rowat AC, Brief E, et al. Universal behavior of membranes with sterols. Biophys J. 2006;90:1639–1649. doi: 10.1529/biophysj.105.067652 16326903 PMC1367315

[183] Romanauska A, Köhler A. Lipid saturation controls nuclear envelope function. Nat Cell Biol. 2023;25:1290–1302. doi: 10.1038/s41556-023-01207-8 37591950 PMC10495262

[184] Zimmerli CE, Allegretti M, Rantos V, et al. Nuclear pores dilate and constrict in cellulo. Science. 2021;374:eabd9776. doi: 10.1126/science.abd9776 34762489

[185] Elosegui-Artola A, Andreu I, Beedle AEM, et al. Force triggers YAP nuclear entry by regulating transport across nuclear pores. Cell. 2017;171:1397–1410.e14. doi: 10.1016/j.cell.2017.10.008 29107331

[186] Andreu I, Granero-Moya I, Chahare NR, et al. Mechanical force application to the nucleus regulates nucleocytoplasmic transport. Nat Cell Biol. 2022;24:896–905. doi: 10.1038/s41556-022-00927-7 35681009 PMC7614780

[187] Selezneva A, Gibb AJ, Willis D. The nuclear envelope as a regulator of immune cell function. Front Immunol. 2022;13:840069. doi: 10.3389/fimmu.2022.840069. PMC9226455 35757775

[188] Alberts B, Johnson A, Lewis J, et al. Chromosomal DNA and its packaging in the chromatin fiber. In: Molecular biology of the cell. 4th ed. New York: Garland Science; 2002. Available from: https://www.ncbi.nlm.nih.gov/books/NBK26834/

[189] Creyghton MP, Cheng AW, Welstead GG, et al. Histone H3K27ac separates active from poised enhancers and predicts developmental state. PNAS. 2010;107(50):21931–21936. doi: 10.1073/pnas.1016071107 PMC3003124 21106759

[190] Maeshima K, Iida S, Shimazoe MA, et al. Is euchromatin really open in the cell? Trends Cell Biol. 2024;34:7–17. doi: 10.1016/j.tcb.2023.05.007 37385880

[191] Fukuda K, Shimi T, Shimura C, et al. Epigenetic plasticity safeguards heterochromatin configuration in mammals. Nucleic Acids Res. 2023;51(12):6190–6207. doi: 10.1093/nar/gkad387 37178005 PMC10325917

[192] Boros J, Arnoult N, Stroobant V, et al. Polycomb repressive complex 2 and H3K27me3 cooperate with H3K9 methylation to maintain heterochromatin protein 1α at chromatin. Mol Cell Biol. 2014;34(19):3662–3674. doi: 10.1128/MCB.00205-14 25047840 PMC4187721

[193] Towbin BD, Gonzalez-Sandoval A, Gasser SM. Mechanisms of heterochromatin subnuclear localization. Trends Biochem Sci. 2013;38:356–363. doi: 10.1016/j.tibs.2013.04.004 23746617

[194] Senapati S, Irshad IU, Sharma AK, et al. Predicting gene expression changes from chromatin structure modification. NPJ Syst Biol Appl. 2025;11(1):34. doi: 10.1038/s41540-025-00510-4 40234426 PMC12000410

[195] Stephens AD, Liu PZ, Banigan EJ, et al. Chromatin histone modifications and rigidity affect nuclear morphology independent of lamins. Mol Biol Cell. 2018;29(2):220–233. doi: 10.1091/mbc.E17-06-0410 29142071 PMC5909933

[196] Pagliara S, Franze K, Cr M, et al. Auxetic nuclei in embryonic stem cells exiting pluripotency. Nat Mater. 2014;13(6):638–644. doi: 10.1038/nmat3943 24747782 PMC4283157

[197] Strom AR, Biggs RJ, Banigan EJ, et al. Hp1α is a chromatin crosslinker that controls nuclear and mitotic chromosome mechanics. Elife. 2021;10:e63972. doi: 10.7554/eLife.63972 34106828 PMC8233041

[198] Nava MM, Miroshnikova YA, Biggs LC, et al. Heterochromatin-driven nuclear softening protects the genome against mechanical stress-induced damage. Cell. 2020;181(4):800–817.e22. doi: 10.1016/j.cell.2020.03.052 32302590 PMC7237863

[199] Schreiner SM, Koo PK, Zhao Y, et al. The tethering of chromatin to the nuclear envelope supports nuclear mechanics. Nat Commun. 2015;6(1):7159. doi: 10.1038/ncomms8159 26074052 PMC4490570

[200] Attar AG, Paturej J, Os S, et al. Peripheral heterochromatin tethering is required for chromatin-based nuclear mechanical response. Nucleic Acids Res. 2025;53(15):gkaf763. doi: 10.1093/nar/gkaf763 40823810 PMC12359041

[201] Jacobson EC, Perry JK, Long DS, et al. Migration through a small pore disrupts inactive chromatin organization in neutrophil-like cells. BMC Biol. 2018;16:142. doi: 10.1186/s12915-018-0608-2 30477489 PMC6257957

[202] Patta I, Zand M, Lee L. Nuclear morphology is shaped by loop-extrusion programs. Nature. 2024;627(8002):196–203. doi: 10.1038/s41586-024-07086-9 38355805 PMC11052650

[203] Alon R, Kassner PD, Carr MW, et al. The integrin VLA-4 supports tethering and rolling in flow on VCAM-1. J Cell Biol. 1995;128(6):1243–1253. doi: 10.1083/jcb.128.6.1243 7534768 PMC2120426

[204] Zhang X, Cook PC, Zindy E, et al. Integrin α4β1 controls G9a activity that regulates epigenetic changes and nuclear properties required for lymphocyte migration. Nucleic Acids Res. 2016;44(7):3031–3044. doi: 10.1093/nar/gkv1348 26657637 PMC4838336

[205] Taverna SD, Li H, Ruthenburg AJ, et al. How chromatin-binding modules interpret histone modifications: lessons from professional pocket pickers. Nat Struct Mol Biol. 2007;14:1025–1040. doi: 10.1038/nsmb1338 17984965 PMC4691843

[206] Erdel F, Rademacher A, Vlijm R, et al. Mouse heterochromatin adopts digital compaction states without showing hallmarks of HP1-driven liquid-liquid phase separation. Mol Cell. 2020;78(2):236–249.e7. doi: 10.1016/j.molcel.2020.02.005 32101700 PMC7163299

[207] Erdel F, Rippe K. Formation of chromatin subcompartments by phase separation. Biophys J. 2018;114(10):2262–2270. doi: 10.1016/j.bpj.2018.03.011 29628210 PMC6129460

[208] Strom AR, Emelyanov AV, Mir M, et al. Phase separation drives heterochromatin domain formation. Nature. 2017;547(7662):241–245. doi: 10.1038/nature22989 28636597 PMC6022742

[209] Sabari BR, Dall’agnese A, Young RA. Biomolecular condensates in the nucleus. Trends Biochem Sci. 2020;45:961–977. doi: 10.1016/j.tibs.2020.06.007 32684431 PMC7572565

[210] Sabari BR, Dall’agnese A, Boija A, et al. Coactivator condensation at super-enhancers links phase separation and gene control. Science. 2018;361(6400):eaar3958. doi: 10.1126/science.aar3958 29930091 PMC6092193

[211] Shin Y, Chang Y-C, Lee DSW, et al. Liquid nuclear condensates mechanically sense and restructure the genome. Cell. 2018;175(6):1481–1491.e13. doi: 10.1016/j.cell.2018.10.057 30500535 PMC6724728

[212] Quail T, Golfier S, Elsner M, et al. Force generation by protein–DNA co-condensation. Nat Phys. 2021;17(9):1007–1012. doi: 10.1038/s41567-021-01285-1

[213] Negri ML, D’Annunzio S, Vitali G, et al. May the force be with you: nuclear condensates function beyond transcription control: potential nongenetic functions of nuclear condensates in physiological and pathological conditions. Bioessays. 2023;45(10):e2300075. doi: 10.1002/bies.202300075 37530178

[214] Fasciani A, D’Annunzio S, Poli V, et al. Mll4-associated condensates counterbalance Polycomb-mediated nuclear mechanical stress in Kabuki syndrome. Nat Genet. 2020;52(12):1397–1411. doi: 10.1038/s41588-020-00724-8 33169020 PMC7610431

[215] Damodaran K, Venkatachalapathy S, Alisafaei F, et al. Compressive force induces reversible chromatin condensation and cell geometry-dependent transcriptional response. MBoC. 2018;29(25):3039–3051. doi: 10.1091/mbc.E18-04-0256 30256731 PMC6333178

[216] Hsia C-R, McAllister J, Hasan O, et al. Confined migration induces heterochromatin formation and alters chromatin accessibility. iScience. 2022;25(9):104978. doi: 10.1016/j.isci.2022.104978 36117991 PMC9474860

[217] Zhao JZ, Xia J, Brangwynne CP. Chromatin compaction during confined cell migration induces and reshapes nuclear condensates. Nat Commun. 2024;15(1):9964. doi: 10.1038/s41467-024-54120-5 39557835 PMC11574006

[218] Todorovski V, McCluggage F, Li Y, et al. Confined environments induce polarized paraspeckle condensates. Commun Biol. 2023;6:145. doi: 10.1038/s42003-023-04528-4 36737664 PMC9898560

[219] Hovet O, Nahali N, Halaburkova A, et al. Nuclear mechano-confinement induces geometry-dependent HP1α condensate alterations. Commun Biol. 2025;8(1):308. doi: 10.1038/s42003-025-07732-6 40000755 PMC11862009

[220] Kodali S, Sands CM, Guo L, et al. Biomolecular condensates in immune cell fate. Nat Rev Immunol. 2025;25(6):445–459. doi: 10.1038/s41577-025-01130-z 39875604 PMC12133415

[221] Wang Y, Zolotarev N, Yang C-Y, et al. A prion-like domain in transcription factor EBF1 promotes phase separation and enables B cell programming of progenitor chromatin. Immunity. 2020;53(6):1151–1167.e6. doi: 10.1016/j.immuni.2020.10.009 33159853

[222] Jia P, Li X, Wang X, et al. Zmynd8 mediated liquid condensates spatiotemporally decommission the latent super-enhancers during macrophage polarization. Nat Commun. 2021;12(1):6535. doi: 10.1038/s41467-021-26864-x 34764296 PMC8586003

[223] Goldman N, Chandra A, Johnson I, et al. Intrinsically disordered domain of transcription factor TCF-1 is required for T cell developmental fidelity. Nat Immunol. 2023;24(10):1698–1710. doi: 10.1038/s41590-023-01599-7 37592014 PMC10919931

[224] Tang B, Wang X, He H, et al. Aging-disturbed FUS phase transition impairs hematopoietic stem cells by altering chromatin structure. Blood. 2024;143(2):124–138. doi: 10.1182/blood.2023020539 37748139

[225] Wang XQD, Fan D, Han Q, et al. Mutant NPM1 hijacks transcriptional hubs to maintain pathogenic gene programs in acute myeloid leukemia. Cancer Discov. 2023;13(3):724–745. doi: 10.1158/2159-8290.CD-22-0424 36455589 PMC9975662

[226] Shin J-Y, Worman HJ. Molecular pathology of laminopathies. Annu Rev Pathol Mech Dis. 2022;17(1):159–180. doi: 10.1146/annurev-pathol-042220-034240 PMC8881990 34672689

[227] Martins F, Sousa J, Pereira CD, et al. Nuclear envelope dysfunction and its contribution to the aging process. Aging Cell. 2020;19(5):e13143. doi: 10.1111/acel.13143 32291910 PMC7253059

[228] Paganelli F, Poli A, Truocchio S, et al. At the nucleus of cancer: how the nuclear envelope controls tumor progression. MedComm (2020). 2025;6:e70073. doi: 10.1002/mco2.70073 39866838 PMC11758262

[229] de Faria RC, Gonzalo S. Sterile inflammation in laminopathies. Eur J Cell Biol. 2025;104:151512. doi: 10.1016/j.ejcb.2025.151512 40897109 PMC13249109

[230] Li X, Li C, Zhang W, et al. Inflammation and aging: signaling pathways and intervention therapies. Sig Transduct Target Ther. 2023;8:239. doi: 10.1038/s41392-023-01502-8 PMC10248351 37291105

[231] Singh N, Baby D, Rajguru JP, et al. Inflammation and cancer. Ann Afr Med. 2019;18:121–126. doi: 10.4103/aam.aam_56_18 31417011 PMC6704802

[232] Panja N, Maji S, Choudhuri S, et al. 3D bioprinting of human hollow organs. AAPS PharmSciTech. 2022;23:139. doi: 10.1208/s12249-022-02279-9 35536418 PMC9088731

[233] Ramsperger AFRM, Narayana VKB, Gross W, et al. Environmental exposure enhances the internalization of microplastic particles into cells. Sci Adv. 2020;6:eabd1211. doi: 10.1126/sciadv.abd1211 33298447 PMC7725476

[234] Ruiz-Fernandez MJA, Jiang J, Mortazavi A, et al. Hijacked immune cells traverse microenvironmental barriers by positioning and pushing their intracellular parasite cargo. BioRxiv. 2024. doi: 10.1101/2024.05.03.592378

[235] Lee LM, Liu AP. The application of micropipette aspiration in molecular mechanics of single cells. J Nanotechnol Eng Med. 2014;5(4):0408011–6. doi: 10.1115/1.4029936 26155329 PMC4476029

[236] Hochmuth RM. Micropipette aspiration of living cells. J Biomech. 2000;33(1):15–22. doi: 10.1016/S0021-9290(99)00175-X 10609514

[237] Dahl KN, Engler AJ, Pajerowski JD, et al. Power-law rheology of isolated nuclei with deformation mapping of nuclear substructures. Biophys J. 2005;89(4):2855–2864. doi: 10.1529/biophysj.105.062554 16055543 PMC1366783

[238] Rowat AC, Lammerding J, Ipsen JH. Mechanical properties of the cell nucleus and the effect of emerin deficiency. Biophys J. 2006;91(12):4649–4664. doi: 10.1529/biophysj.106.086454 16997877 PMC1779937

[239] Irianto J, Xia Y, Pfeifer CR, et al. As a nucleus enters a small pore, chromatin stretches and maintains integrity, even with DNA breaks. Biophys J. 2017;112(3):446–449. doi: 10.1016/j.bpj.2016.09.047 28341535 PMC5300774

[240] Irianto J, Pfeifer CR, Bennett RR, et al. Nuclear constriction segregates mobile nuclear proteins away from chromatin. Mol Biol Cell. 2016;27(25):4011–4020. doi: 10.1091/mbc.E16-06-0428 27798234 PMC5156542

[241] Dos Santos Á, Rehfeldt F, Toseland CP. Measuring nuclear mechanics with atomic force microscopy. Methods Mol Biol. 2022;2476:171–181. doi: 10.1007/978-1-0716-2221-6_13 35635704

[242] Cartagena-Rivera AX, Logue JS, Waterman CM, et al. Actomyosin cortical mechanical properties in nonadherent cells determined by atomic force microscopy. Biophys J. 2016;110(11):2528–2539. doi: 10.1016/j.bpj.2016.04.034 27276270 PMC4906360

[243] Garcia R. Nanomechanical mapping of soft materials with the atomic force microscope: methods, theory and applications. Chem Soc Rev. 2020;49(16):5850–5884. doi: 10.1039/D0CS00318B 32662499

[244] Liu H, Wen J, Xiao Y, et al. In situ mechanical characterization of the cell nucleus by atomic force microscopy. ACS Nano. 2014;8(4):3821–3828. doi: 10.1021/nn500553z 24673613

[245] Català-Castro F, Schäffer E, Krieg M. Exploring cell and tissue mechanics with optical tweezers. J Cell Sci. 2022;135(15):jcs259355. doi: 10.1242/jcs.259355 35942913

[246] Català-Castro F, Venturini V, Ortiz-Vásquez S, et al. Direct Force Measurements of Subcellular Mechanics in Confinement using Optical Tweezers. J Vis Exp. 2021;(174). doi: 10.3791/62865-v 34542528

[247] Heuzé ML, Collin O, Terriac E, et al. Cell migration in confinement: a micro-channel-based assay. Methods Mol Biol. 2011;769:415–434. doi: 10.1007/978-1-61779-207-6_28 21748692

[248] Vargas P, Chabaud M, Thiam H-R, et al. Study of dendritic cell migration using micro-fabrication. J Immunol Methods. 2016;432:30–34. doi: 10.1016/j.jim.2015.12.005 26684937

[249] Sáez PJ, Barbier L, Attia R, et al. Leukocyte migration and deformation in collagen gels and microfabricated constrictions. Methods Mol Biol. 2018;1749:361–373. doi: 10.1007/978-1-4939-7701-7_26 29526010

[250] Isermann P, Davidson PM, Sliz JD, et al. Assays to measure nuclear mechanics in interphase cells. Curr Protoc Cell Biol. 2012; Chapter 22:Unit22.16. 56(1). doi: 10.1002/0471143030.cb2216s56 PMC3605726 22968843

[251] Renkawitz J, Reversat A, Leithner A, et al. Micro-engineered “pillar forests” to study cell migration in complex but controlled 3D environments. Methods Cell Biol. 2018;147:79–91. doi: 10.1016/bs.mcb.2018.07.004 30165964

[252] Davidson PM, Fedorchak GR, Mondésert-Deveraux S, et al. High-throughput microfluidic micropipette aspiration device to probe time-scale dependent nuclear mechanics in intact cells. Lab Chip. 2019;19:3652–3663. doi: 10.1039/c9lc00444k 31559980 PMC6810812

[253] Davidson PM, Sliz J, Isermann P, et al. Design of a microfluidic device to quantify dynamic intra-nuclear deformation during cell migration through confining environments. Integr Biol (Camb). 2015;7:1534–1546. doi: 10.1039/c5ib00200a 26549481 PMC4666765

[254] Le Berre M, Aubertin J, Piel M. Fine control of nuclear confinement identifies a threshold deformation leading to lamina rupture and induction of specific genes. Integr Biol (Camb). 2012;4:1406–1414. doi: 10.1039/c2ib20056b 23038068

[255] Le Berre M, Zlotek-Zlotkiewicz E, Bonazzi D, et al. Methods for two-dimensional cell confinement. Methods Cell Biol. 2014;121:213–229. doi: 10.1016/B978-0-12-800281-0.00014-2. PMID: 24560512. 24560512

[256] Lancaster OM, Le Berre M, Dimitracopoulos A, et al. Mitotic Rounding Alters Cell Geometry to Ensure Efficient Bipolar Spindle Formation. Dev Cell. 2013;25:270–283. doi: 10.1016/j.devcel.2013.03.014 23623611

[257] Velve-Casquillas G, Le Berre M, Piel M, et al. Microfluidic tools for cell biological research. Nano Today. 2010;5:28–47. doi: 10.1016/j.nantod.2009.12.001 21152269 PMC2998071

[258] Maremonti MI, Panzetta V, Dannhauser D, et al. Wide-range viscoelastic compression forces in microfluidics to probe cell-dependent nuclear structural and mechanobiological responses. J R Soc Interface. 2022;19(189):20210880. doi: 10.1098/rsif.2021.0880 35440204 PMC9019521

[259] Kroll J, Ruiz-Fernandez MJA, Braun MB, et al. Quantifying the probing and selection of microenvironmental pores by motile immune cells. Curr Protoc. 2022;2(4):e407. doi: 10.1002/cpz1.407 35384410

[260] Irianto J, Xia Y, Pfeifer CR, et al. DNA damage follows repair factor depletion and portends genome variation in cancer cells after pore migration. Curr Biol. 2017;27:210–223. doi: 10.1016/j.cub.2016.11.049 27989676 PMC5262636

[261] Danielsson BE, George Abraham B, Mäntylä E, et al. Nuclear lamina strain states revealed by intermolecular force biosensor. Nat Commun. 2023;14:3867. doi: 10.1038/s41467-023-39563-6 37391402 PMC10313699

[262] Colom A, Derivery E, Soleimanpour S, et al. A fluorescent membrane tension probe. Nat Chem. 2018;10:1118–1125. doi: 10.1038/s41557-018-0127-3 30150727 PMC6197433

[263] Kabakova I, Zhang J, Xiang Y, et al. Brillouin microscopy. Nat Rev Methods Primers. 2024;4(1):8. doi: 10.1038/s43586-023-00286-z 39391288 PMC11465583

[264] Prevedel R, Diz-Muñoz A, Ruocco G, et al. Brillouin microscopy: an emerging tool for mechanobiology. Nat Methods. 2019;16(10):969–977. doi: 10.1038/s41592-019-0543-3 31548707

[265] Kerdegari S, Passeri AA, Morena F, et al. Contact-free characterization of nuclear mechanics using correlative Brillouin-Raman micro-spectroscopy in living cells. Acta Biomater. 2025;198:291–301. doi: 10.1016/j.actbio.2025.04.009 40189116

[266] Wirtz D. Particle-tracking microrheology of living cells: principles and applications. Annu Rev Biophys. 2009;38(1):301–326. doi: 10.1146/annurev.biophys.050708.133724 19416071

[267] Tseng Y, Lee JSH, Kole TP, et al. Micro-organization and visco-elasticity of the interphase nucleus revealed by particle nanotracking. J Cell Sci. 2004;117(10):2159–2167. doi: 10.1242/jcs.01073 15090601

[268] Delarue M, Brittingham GP, Pfeffer S, et al. mTORC1 controls phase separation and the biophysical properties of the cytoplasm by tuning crowding. Cell. 2018;174:338–349.e20. doi: 10.1016/j.cell.2018.05.042 29937223 PMC10080728

[269] Shu T, Szórádi T, Kidiyoor GR, et al. nucGEMs probe the biophysical properties of the nucleoplasm. BioRxiv. 2021. doi: 10.1101/2021.11.18.469159

